# Expression Patterns and miRNA Regulation of DNA Methyltransferases in Chicken Primordial Germ Cells

**DOI:** 10.1371/journal.pone.0019524

**Published:** 2011-05-03

**Authors:** Deivendran Rengaraj, Bo Ram Lee, Sang In Lee, Hee Won Seo, Jae Yong Han

**Affiliations:** WCU Biomodulation Major, Department of Agricultural Biotechnology and Research Institute for Agriculture and Life Sciences, Seoul National University, Seoul, Korea; Istituto Dermatologico dell'Immacolata, Italy

## Abstract

DNA methylation is widespread in most species, from bacteria to mammals, and is crucial for genomic imprinting, gene expression, and embryogenesis. DNA methylation occurs via two major classes of enzymatic reactions: maintenance-type methylation catalyzed by DNA (cytosine-5-)-methyltransferase (DNMT) 1, and *de novo* methylation catalyzed by DNMT 3 alpha (DNMT3A) and -beta (DNMT3B). The expression pattern and regulation of *DNMT* genes in primordial germ cells (PGCs) and germ line cells has not been sufficiently established in birds. Therefore, we employed bioinformatics, RT-PCR, real-time PCR, and *in situ* hybridization analyses to examine the structural conservation and conserved expression patterns of chicken *DNMT* family genes. We further examined the regulation of a candidate *de novo* DNA methyltransferase gene, c*DNMT3B* by cotransfection of c*DNMT3B* 3′UTR- and c*DNMT3B* 3′UTR-specific miRNAs through a dual fluorescence reporter assay. All c*DNMT* family members were differentially detected during early embryonic development. Of interest, c*DNMT3B* expression was highly detected in early embryos and in PGCs. During germ line development and sexual maturation, c*DNMT3B* expression was reestablished in a female germ cell-specific manner. In the dual fluorescence reporter assay, c*DNMT3B* expression was significantly downregulated by four miRNAs: gga-miR-15c (25.82%), gga-miR-29b (30.01%), gga-miR-383 (30.0%), and gga-miR-222 (31.28%). Our data highlight the structural conservation and conserved expression patterns of chicken *DNMT*s. The miRNAs investigated in this study may induce downregulation of gene expression in chicken PGCs and germ cells.

## Introduction

Methylation represents the addition of a methyl group or replacement of an atom by a methyl group in a substrate. The addition of a methyl group to the 5-position of the cytosine nucleotide in the sequence CpG by catalyzing enzymes is called DNA methylation. DNA methylation is widespread in most species, from bacteria to mammals. In mammals, DNA methylation is crucial for normal development, most likely due to its importance in genomic imprinting, X-chromosome inactivation, chromatin modification, silencing of endogenous retroviruses, gene expression, and embryo development [1], [2]. DNA methylation is primarily negative during zygote formation, and is established through cell division during embryonic development [2]. The occurrence of DNA methylation is closely connected with chromatin organization by histone acetylation and methylation. Histone acetyltransferases (HDACs) mainly catalyze the enzymatic reactions of histone acetylation [3]. DNA methylation occurs via two major classes of enzymatic reaction: maintenance-type methylation and *de novo* methylation. Maintenance-type methylation activity involves the maintenance of methylation patterns in the daughter strands of every DNA replication cycle. *De novo* methylation activity involves the recognition and transfer of methyl groups to unmethylated DNA [4].

There are three enzymes in the DNA (cytosine-5-)-methyltransferase (DNMT) family: DNMT1, DNMT 3 alpha (DNMT3A), and DNMT 3 beta (DNMT3B). All catalyze DNA methylation activity. DNMT1 is a member of the maintenance-type methyltransferase family, which is responsible for the maintenance of DNA methylation patterns [5]. DNMT3A and the closely related DNMT3B are *de novo* methyltransferases, which are responsible for the establishment of new methylation patterns [2], [5]. DNMT1 and DNMT3A expressions are ubiquitous, whereas DNMT3B is expressed at a low level in most tissues except the testis, pancreas, thyroid, and bone marrow. DNA methylation and DNMT family proteins play global functions in vertebrate species. DNMTs act as potential molecular targets in cancer therapy. Overexpression of DNMTs has been shown to influence tumor cell resistance to cytotoxicity of oxidative stress [6]. DNMT1 is associated with the perpetuation of fibroblast activation and fibrogenesis in the kidney [7]. DNMT1 and DNMT3A are required for neuronal synaptic plasticity, learning, and memory [8].

Compared to mammalian species [9], the expression pattern and regulation of *DNMT* genes during germ line development has not been sufficiently established in birds. In this study, we examined the conservation and functional domains of cDNMT family proteins using bioinformatics analysis, and further examined the conserved expression patterns of c*DNMT* family genes during early embryonic development, germ line development, and sexual maturation of testis and ovaries using reverse transcription PCR (RT-PCR), quantitative real-time PCR (qRT-PCR), and *in situ* hybridization analyses. To examine the regulation of the candidate *de novo* DNA methyltransferase gene c*DNMT3B* at the post-transcriptional level, we performed cotransfection analysis using c*DNMT3B* 3′UTR- (3 prime untranslated regions) and c*DNMT3B* 3′UTR-specific microRNAs (miRNAs). All c*DNMT* family members were differentially detected during early embryonic development. Of interest, c*DNMT3B* expression was highly detected in early embryos, primordial germ cells (PGCs), and germ cells at least until embryonic day E14.5. After hatching, c*DNMT3B* expression was reestablished in a female germ cell-specific manner. In the dual fluorescence reporter assay, c*DNMT3B* expression was significantly downregulated by all miRNAs examined. The miRNAs investigated in this study may induce downregulation of gene expression in chicken PGCs and germ cells.

## Materials and Methods

### Experimental animals and animal care

The care and experimental use of White Leghorn chickens were approved by the Institute of Laboratory Animal Resources, Seoul National University (SNU-070823-5), Korea. Chickens were maintained according to a standard management program at the University Animal Farm, Seoul National University. The procedures for animal management, reproduction, and embryo manipulation adhered to the standard operating protocols of our laboratory.

### Sex determination

Freshly laid eggs were incubated with intermittent rocking at 37°C under 60–70% relative humidity. Sex was determined on embryonic day E2.5. Approximately 0.2 µL of embryonic blood was collected from the dorsal aorta, diluted in 15 µL of 1× phosphate buffered saline (PBS, pH 7.4), and boiled at 94°C for 10 min to prepare the DNA template for PCR. Each 20-µL PCR reaction contained 2 µL of DNA template, 2 µL of PCR buffer, 1.6 µL of 2.5-mM dNTP mixture, 10 pmol of each forward and reverse primer of chicken W chromosome (F: 5′-CTA TGC CTA CCA CAT TCC TAT TTG C-3′ and R: 5′-AGC TGG ACT TCA GAC CAT CTT CT-3′), and 1 unit of Taq DNA polymerase. The thermal conditions for 35 cycles were 94°C for 30 s, 66°C for 30 s, and 72°C for 30 s. Female sex was identified based on the strong bands detected in the agarose gel after separation of PCR products by gel electrophoresis.

### Sample collection

We collected whole embryos at EG&K [10] stage X (freshly laid eggs), H&H [11] stage 3 (12-h incubation), H&H stage 6 (24-h incubation), and H&H stage 12 (48-h incubation). We collected blood PGCs (bPGCs) on E2.5, and gonadal PGCs (gPGCs) and gonadal stromal cells (GSCs) on E6.5 by magnetic-activated cell sorting (MACS) [12]. bPGCs were cultured (hereafter known as cPGCs) up to passage 30, as previously described [13]. Apart from the whole embryos and cell samples, we collected embryonic gonads on E4.5; embryonic brains, kidneys, livers, stomachs, muscles, lungs, and gonads from male and female embryos on E6.5; gonads from male and female embryos on E8.5, E9.5, E10.5, E11.5, E12.5, E13.5, E14.5 and E15.5; and testes and ovaries at 1 day, 12 weeks, and 25 weeks of age. Total RNA was extracted from three batches of the aforementioned samples using Trizol reagent (Invitrogen, Carlsbad, CA, USA) according to the manufacturer's protocols. Approximately 1 µg of Oligo(dT)_20_-primed total RNA from each sample was reverse transcribed with the Superscript III First-Strand Synthesis System (Invitrogen) according to the manufacturer's protocols. All cDNA samples were diluted to 10% and used for RT-PCR, qRT-PCR, and subcloning experiments. Another batch of limited samples were directly used/frozen in liquid nitrogen for the *in situ* hybridization experiment.

### Analysis of structural features and conservation of cDNMT family proteins

The protein sequences of chicken DNMT family members were obtained from a BLAST search of the Chicken Genome Database at the National Center for Biotechnology Information (NCBI). The protein sequences of chicken DNMT1 (NP_996835), DNMT3A (NP_001020003), and DNMT3B (NP_001019999) were used to search for homologous DNMT family members in all vertebrate species using the NCBI BLASTP search engine. We retrieved all DNMT family protein sequences from at least nine other vertebrate species, including human, chimpanzee, cattle, pig, horse, rat, mouse, opossum, and zebrafish (see Table 1 for GenBank accession numbers). The percent identities and conservation of chicken DNMT family protein sequences with other vertebrate DNMT family protein sequences were analyzed using the NCBI BLASTP and CLUSTAL X programs, respectively. The conserved functional domains of chicken DNMT family protein sequences were identified using the Pfam-A family matrices [14].

**Table 1:** GenBank accession numbers of DNMT family proteins of different vertebrate species obtained by BLASTP searches.

Species	DNMT1	DNMT3A	DNMT3B
Chicken	NP_996835	NP_001020003	NP_001019999
Human	NP_001124295	NP_783328	NP_008823
Chimpanzee	XP_001163364	XP_001148246	XP_514580
Cattle	NP_872592	AAP75901	NP_861529
Pig	NP_001027526	NP_001090906	NP_001155876
Horse	XP_001916472	XP_001503030	XP_001916549
Rat	NP_445806	NP_001003958	NP_001003959
Mouse	NP_034196	NP_031898	NP_001003961
Opossum	NP_001028141	XP_001380132	XP_001362485
Zebrafish	NP_571264	NP_001018150	NP_001020621

### RT-PCR analysis

We performed RT-PCR analysis to examine the tissue-specific expression of c*DNMT1*, c*DNMT3A*, and c*DNMT3B* during early embryonic development. The cDNA from stages X, 3, 6, and 12 embryos; bPGCs, cPGCs, gPGCs, and GSCs; and the brains, kidneys, livers, stomachs, muscles, lungs, and gonads of male and female embryos at E6.5 were amplified using c*DNMT1*, c*DNMT3A*, c*DNMT3B*, and chicken glyceraldehyde-3-phosphate dehydrogenase (c*GAPDH*, NM_204305)-specific primers (Table 2). Each 20-µL PCR reaction mix contained 2 µL of cDNA, 2 µL of PCR buffer, 1.6 µL of 2.5 mM dNTP mixture, 10 pmol of each forward and reverse primer, and 1 unit of Taq DNA polymerase. PCR was performed with an initial incubation at 94°C for 5 min, followed by 30 cycles at 94°C for 30 s, 60°C for 30 s, and 72°C for 30 s. The reaction was terminated by a final incubation at 72°C for 7 min.

**Table 2:** Primers used for c*DNMT* family members, c*GAPDH*, c*DAZL* and c*SYCP3*.

Gene	Accession no.	*Primer sequences (5′-3′)	Size (bps)
c*DNMT1*	NM_206952	F : CTGAGATGCCCTCCCCCAAGR : GTCCTCCCGTCGTCCTCCAC	454
		F : TGTCCATCTTCGACGCCAACR : CATAGATGGGCTTCACGGCA	174
c*DNMT3A*	NM_001024832	F : GCAAGCAGCAGAGCAGGGAAR : CCACCAACAGGTCCACGCA	577
		F : GGGTGAGCGACAAAAGGGACR : TGGAGTTGGAGCGAGTGGTG	234
c*DNMT3B*	NM_001024828	F : GAACCCAGCCACCTTCCACCR : AGTGATGTTGCCCTCGTGCC	547
		F : ACCAGCCAAGAGGAGACCCAR : TGGCGAGCGAGAGGTCATTA	269
c*GAPDH*	NM_204305	F : CACAGCCACACAGAAGACGGR : CCATCAAGTCCACAACACGG	443
		F : CCGTGTTGTGGACTTGATGGR : GAGGAGTGGGGGAGACAGAA	175
c*DAZL*	NM_204218	F: CGTCAACAACCTGCCAAGGAR: TTCTTTGCTCCCCAGGAACC	540
c*SYCP3*	XM_416330	F: GCAGAAAGCAGAGGAACAGGAGGR: TGGACTGAAGAGACTTGCGAACA	281

*First primer pairs were used for RT-PCR analysis and cRNA probe synthesis, and second primer pairs were used for qRT-PCR analysis. c*DAZL* primer pairs were used for cRNA probe synthesis only. c*SYCP3* primer pairs were used for qRT-PCR only.

### qRT-PCR analysis

We performed qRT-PCR analysis to examine the relative quantification of the expression level of c*DNMT1*, c*DNMT3A*, and c*DNMT3B* during early embryonic development and germ line development. The cDNA from stages X, 3, 6, and 12 embryos; bPGCs, cPGCs, gPGCs, and GSCs; male and female gonads at E6.5, E8.5, E10.5, E12.5, and E14.5; and testes and ovaries of chickens at 1 day, 4 weeks, 12 weeks, and 25 weeks of age were amplified with the forward and reverse primers of c*DNMT1*, c*DNMT3A*, c*DNMT3B*, and c*GAPDH* (Table 2). Quantification was performed using an iCycler Real Time PCR Detection System (Bio-Rad Laboratories, Hercules, CA, USA). Each 20-µL PCR reaction mix contained 2 µL of cDNA, 2 µL of PCR buffer, 1.6 µL of 2.5 mM dNTP mixture, 10 pmol of each forward and reverse primer, 1 µL of 20× Eva green (Biotium Inc., Hayward, CA, USA), and 1 unit of Taq DNA polymerase. The reaction was carried out in optical 96-well standard plates (Applied Biosystems Inc., Foster City, CA, USA). PCR was performed with an initial incubation at 94°C for 3 min, followed by 40 cycles at 94°C for 30 s, 60°C for 30 s, and 72°C for 30 s. The reaction was terminated by a final incubation at the dissociation temperatures. Furthermore, expression of c*DNMT3B* during limited time points (E8.5, E10.5, E12.5, E14.5 and 1-day) of meiotic stages were compared with a meiosis specific gene chicken synaptonemal complex protein 3 (c*SYCP3*, please see table 2 for primers). The relative quantification of c*DNMT1*, c*DNMT3A*, c*DNMT3B* and c*SYCP3* expression was calculated using the 2^−ΔΔCt^ method after the threshold cycle (*C*t) was normalized with the *C*t of c*GAPDH*.

We performed miRNA qRT-PCR analysis to examine the relative quantification of the expression level of gga-miR-15c, gga-miR-29b, gga-miR-383 and gga-miR-222 during germ line development. First strand cDNA was synthesized from total RNA (1 µg) of male and female gonads on E9.5, E10.5, E11.5, E12.5, E13.5, E14.5 and E15.5 using the miRNA first strand cDNA synthesis kit (Stratagene, Santa Clara, CA, USA). To elongate the miRNAs, total RNAs were first treated with *E. coli* poly-A polymerase (PAP) to generate a poly-A tail at the 3′-end of each RNA molecule. Following polyadenylation, cDNAs were synthesized using the RT adaptor primer. qRT-PCR analysis for the complete miRNA first strand cDNAs was performed using the High-Specificity miRNA QPCR Core Reagent Kit (Stratagene). Each 25-µL PCR reaction mix contained 4 µL of miRNA cDNA, 2.5 µL of 10× core PCR buffer, 2.75 µL of 50 mM MgCl2, 10 µL of 20 mM dNTPs, 1.25 µL of 20× Eva Green, 1.0 µL of 3.125 µM universal reverse primer, 1.0 µL of 3.125 µM miRNA-specific forward primer and 0.5 µL of High-Specificity polymerase. Each miRNA forward primer was designed according to the guidelines provided by Stratagene (http://www.stratagene.com/miRNAguide). The threshold cycle of miRNA expression were normalized with chicken snoRNA (endogenous control). Please see table 3 for miRNA-specific primers.

**Table 3:** qRT-PCR primers used for gga-miR-15c, gga-miR-29b, gga-miR-383, gga-miR-222 and SnoRNA.

miRNA	Forward primer sequences (5′-3′)
gga-miR-15c	TAGCAGCACATCATGGTTTG
gga-miR-29b	TAGCACCATTTGAAATCAGT
gga-miR-383	AGATCAGAAGGTGATTGTGGCT
gga-miR-222	AGCTACATCTGGCTACTGGGTCTC
SnoRNA	GGGATGTAAAAAAATACTTGCTATC

### 5-methylcytosine staining

5-methylcytosine staining (5 meC) was performed to examine the DNA methylation pattern in PGCs as previously described [15]. Briefly, MACS-sorted PGCs at E2.5, E4.5, and E6.5 were mounted on glass slides treated with 3-aminopropyltriethoxysilane (APES, Sigma-Aldrich, St. Louis, MO, USA) and then fixed with 3.7% (w/v) paraformaldehyde in 1× PBS for 10 min. The cells were washed three times for 5 min each in 1× PBS and permeabilized with 0.5% (v/v) Triton X-100 and 1% (w/v) bovine serum albumin (BSA) in PBS for 30 min. The cells were then washed with 1× PBS, treated with 4N HCl for 20 min, and blocked with 0.1% Triton X-100 and 1% BSA in 1× PBS for 30 min. The cells were incubated with 5 meC antibody (abCAM, Cambridge, UK) and diluted in blocking buffer (1∶200) at 4°C overnight. After primary antibody incubation, cells were washed in 1× PBS and incubated with Alexa 488 dye-conjugated secondary antibodies (Molecular Probes, Carlsbad, CA, USA) for 1 h at room temperature in the dark. Finally, the cells were mounted with ProLongH Gold antifade reagent with 4′-6-diamidino-2-phenylindole (DAPI, Invitrogen) and imaged with a confocal laser microscope (Carl Zeiss, Oberkochen, Germany).

### cRNA probes

To prepare cRNA probes for c*DNMT1*, c*DNMT3A*, c*DNMT3B*, and c*DAZL* (positive control), the cDNA from gPGCs at E6.5 was amplified with the respective primers (Table 2). PCR products were loaded onto 1% agarose gels and separated by gel electrophoresis at 5 v/cm for 30 min. Size-corrected DNAs were subcloned into a pGEM-T plasmid vector (Promega, Madison, WI, USA) and transformed to *Escherichia coli* strain DH5α. The sequences of recombinant plasmids containing each gene were verified using the Automated DNA sequencer 3730×l (Applied Biosystems), and then the recombinant plasmids were amplified using T7- and SP6-specific primers (T7: 5′-TGT AAT ACG ACT CAC TAT AGG G-3′ and SP6: 5′-CTA TTT AGG TGA CAC TAT AGA AT-3′) to prepare templates for labeling cRNA probes. Digoxigenin (DIG)-labeled cRNA probes of c*DNMT1*, c*DNMT3A*, c*DNMT3B*, and c*DAZL* were prepared using a DIG RNA labeling kit (Roche Diagnostics, Indianapolis, IN, USA) by an *in vitro* transcription method.

### *In situ* hybridization of whole mount and cryosections

The expression levels of c*DNMT1*, c*DNMT3A*, and c*DNMT3B* mRNA during early embryonic development, PGC differentiation, germ line development, and sexual maturation were examined at limited time points from stage X to 25 weeks of age by *in situ* hybridization. The expression patterns of c*DNMT1*, c*DNMT3A*, and c*DNMT3B* were compared to that of the germ line-specific gene c*DAZL* at E4.5 and E6.5. Whole embryos (stages X, 6, and 12) collected in Petri dishes, cryosections of gonads, testes, and ovaries (E4.5 to 25 weeks old) mounted on APES-treated glass slides were fixed with 4% (w/v) paraformaldehyde in 1× PBS. The samples were permeabilized with 1% (v/v) Triton X-100 and incubated in a prehybridization mixture containing 50% formamide, 25% 20× standard saline citrate (SSC: 150 mM NaCl and 15 mM sodium citrate; pH 7.0), and 25% diethylpyrocarbonate (DEPC)-treated distilled water. After prehybridization, samples were incubated in a hybridization mixture containing 10% dextran sulfate sodium, 0.02% BSA, 250 µg/mL yeast tRNA, and DIG-labeled cRNA probes in a prehybridization mixture for 18 h at 55°C. The samples were washed for stringency in a series of solutions as previously described [16]. Nonspecific binding was blocked with a 1% (w/v) blocking reagent before the samples were incubated with a sheep anti-DIG-AP antibody (Roche) for 12 h at 4°C. The mRNA signals were visualized as a dark brown color using a substrate solution containing nitroblue tetrazolium (NBT), 5-bromo-4-chloro-3-indolyl phosphate (BCIP), and levamisole. After signal development, the sections were counterstained with 1% (w/v) methyl green (Sigma-Aldrich). Photographs of whole mount embryos were taken with a Stereoscopic Zoom Microscope SMZ1000 (Nikon Corporation, Tokyo, Japan). Photographs of sections were taken with a Zeiss Axiophot light microscope (Carl Zeiss).

### Regulation of *cDNMT3B* using 3′UTR target miRNAs

To examine the regulation of c*DNMT3B* expression, we selected four miRNAs including gga-miR-15c (5′-UAG CAG CAC AUC AUG GUU UGU A-3′), gga-miR-29b (5′-UAG CAC CAU UUG AAA UCA GUG UU-3′), gga-miR-383 (5′-AGA UCA GAA GGU GAU UGU GGC U-3′), and gga-miR-222 (5′-AGC UAC AUC UGG CUA CUG GGU CUC-3′) specific to the 3′UTR region of c*DNMT3B* from miRDB, a microRNA target prediction and functional annotation database [17]. c*DNMT3B* 3′UTR and *cDNMT3B* 3′UTR mutants were cloned into a pcDNA3 plasmid encoding enhanced green fluorescence protein (eGFP), and c*DNMT3B* 3′UTR-specific miRNAs were cloned into a pDsRed2-N1 plasmid encoding red fluorescence protein (RFP) under a CMV promoter (Clontech, Palo Alto, CA, USA) for the dual fluorescence reporter assay. Plasmids containing c*DNMT3B* 3′UTR and miRNA were prepared in a 100-mL LB culture of transformed XL1-Blue *E. coli* using an EndoFree Plasmid Maxi kit (Qiagen, Valencia, CA, USA) and cotransfected into 293 FT cells using the transfection procedures provided by Invitrogen. After 48 h of cotransfection, eGFP expression was examined under the microscope, and the relative eGFP expression was analyzed by fluorescence-activated cell sorting (FACS) using BD FACSCalibur (BD Biosciences, San Jose, CA, USA). Furthermore, conservation of c*DNMT3B* 3′UTR region and target miRNAs in chicken, human and mouse were analyzed using the CLUSTAL X programs.

## Results

### Conserved structural features and sequence analysis of cDNMT family proteins

**Figure 1:**
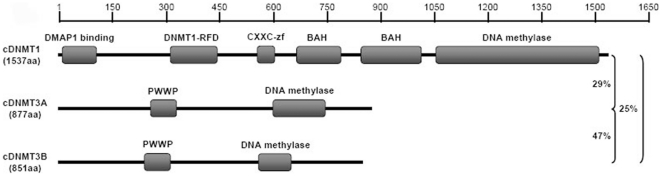
Graphic diagram of the conserved functional domains. The conserved functional domains of cDNMT1, cDNMT3A, and cDNMT3B protein sequences found using the Pfam-A family matrices with default parameters.

The mRNA and protein sequences of chicken *DNMT* family genes were obtained from the NCBI *Gallus gallus* Genome Database. Of these, the mRNA sequence of c*DNMT1* contains an open reading frame of 4614 base pairs (bp) encoding a 1537-amino acid protein. The percent identity of cDNMT1 protein with other vertebrate DNMT1 proteins over the entire alignment indicated significant identities: 82% to opossum; 75% to human, cattle, and horse; 73% to chimpanzee; 71% to rat, mouse, and zebrafish; and 70% to pig. cDNMT1 had significant hits to five different functional domains including DMAP1 (DNMT1-associated protein 1) binding domain (at position 8–100 aa, E-value 3e-16), DNMT1-specific replication foci domain (at position 310–445 aa, E-value 4.1e-38), CXXC zinc finger domain (at position 557–603 aa, E-value 1.5e-14), bromo-adjacent homology (BAH) domains (at positions 667–791 aa and 842–1011 aa, E-values 9.1e-22 and 7.1e-18), and C-5 cytosine-specific DNA methylase domain (at position 1054–1508 aa, E-value 3.8e-48), which is also conserved in all investigated vertebrate DNMT1 proteins (Fig. 1 and Fig. S1). The mRNA sequence of the second member, c*DNMT3A*, contains an open reading frame of 2634 bp encoding an 877-amino acid protein. The percent identity of cDNMT3A protein with other vertebrate DNMT3A proteins was: 93% to pig; 87% to horse and opossum; 86% to human, chimpanzee, cattle, rat, and mouse; and 81% to zebrafish. cDNMT3A had significant hits to two functional domains including the PWWP domain (at position 254–327 aa, E-value 1.4e-23) and C-5 cytosine-specific DNA methylase domain (at position 599–741 aa, E-value 1.7e-12), which is also conserved in other vertebrate DNMT3A proteins (Fig. 1 and Fig. S2). The mRNA and protein length of the third member, c*DNMT3B*, is comparatively shorter than those of other members of the *DNMT* family. c*DNMT3B* mRNA consists of an open reading frame of 2556 bp encoding an 851-amino acid protein. cDNMT3B shares significant identities with other vertebrate DNMT3B proteins for up to 70% to cattle, 67% to opossum, 66% to horse, 64% to human and chimpanzee, 60% to pig, 54% to zebrafish, 45% to rat, and 43% to mouse. Similar to cDNMT3A, cDNMT3B also showed significant hits to the PWWP domain (at position 236–309 aa, E-value 1.9e-20) and C-5 cytosine-specific DNA methylase domain (at position 569–657 aa, E-value 2e-06), which is conserved in all vertebrate DNMT3B proteins (Fig. 1 and Fig. S3). Furthermore, cDNMT1 shares 29% identity with cDNMT3A and 25% identity with cDNMT3B. The identity between cDNMT3A and cDNMT3B is 47% (Fig. 1).

### Expression of cDNMT family members during early embryonic development examined by RT-PCR

**Figure 2:**
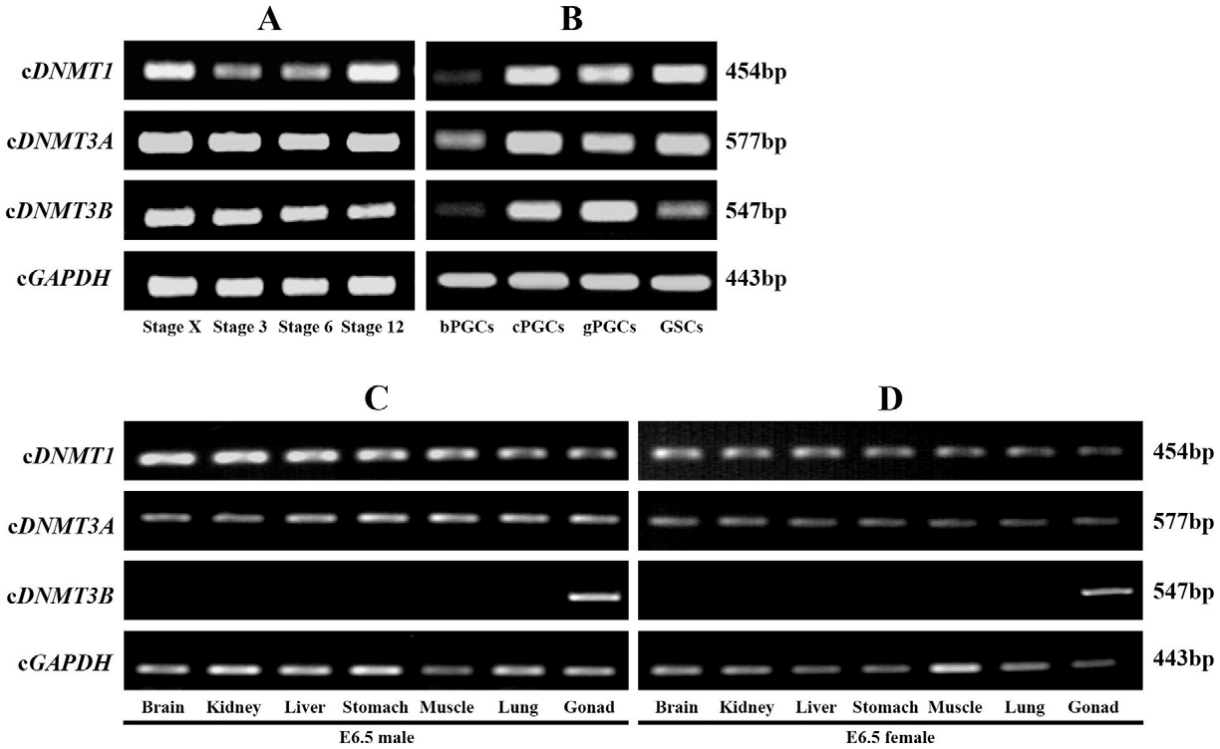
Expression of c*DNMT1*, c*DNMT3A*, and c*DNMT3B* during early embryonic development examined by RT-PCR. cDNA from EG&K stage X, H&H stage 3, stage 6, and stage 12 (2A); blood PGCs (bPGCs), cultured PGCs (cPGCs), gonadal PGCs (gPGCs), and gonadal stromal cells (GSCs) (2B); and the brains, kidneys, livers, stomachs, muscles, lungs, and gonads of male and female embryos at E6.5 (2C, 2D) were amplified with c*DNMT1*, c*DNMT3A*, c*DNMT3B*, and chicken glyceraldehyde 3 phosphate dehydrogenase (c*GAPDH*)-specific primers.

Expression of c*DNMT1*, c*DNMT3A*, and c*DNMT3B* in early embryos at stages X, 3, 6, and 12; bPGCs, cPGCs, gPGCs, and GSCs; and the brains, kidneys, livers, stomachs, muscles, lungs, and gonads of male and female embryos at E6.5 were examined by RT-PCR. c*DNMT1* and c*DNMT3A* showed similar patterns of expression in all tissues/cells examined. During early embryonic development, c*DNMT1* and c*DNMT3A* were detected at a strong level in the embryos at stages X, 3, 6, and 12 (Fig. 2A). Both c*DNMT1* and c*DNMT3A* were detected at low levels in bPGCs compared to cPGCs, gPGCs, and GSCs; however, c*DNMT1* expression was much lower in bPGCs (Fig. 2B). c*DNMT1* and c*DNMT3A* expressions were detected at moderate levels in all somatic and gonadal samples of male and female embryos examined at E6.5 (Fig. 2C–D). c*DNMT3B* expression differed from that of the other members. Expression was strongly detected in all early embryos, cPGCs, gPGCs, and male and female gonads (Fig. 2A–D); weakly detected in bPGCs and GSCs (Fig. 2B); and not detected or barely detected in all somatic tissues of male and female embryos at E6.5 (Fig. 2C–D).

### Expression of cDNMT family members during early embryonic development, germ-line development, and sexual maturation examined by qRT-PCR

**Figure 3:**
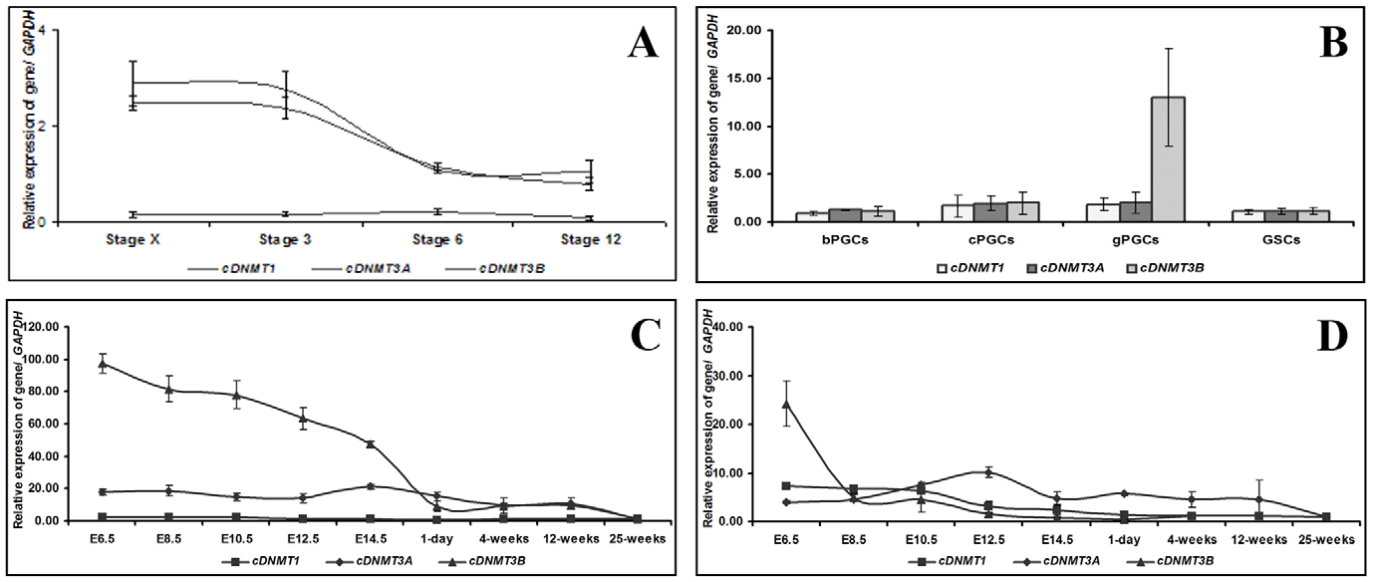
Expression of c*DNMT1*, c*DNMT3A*, and c*DNMT3B* during early embryonic development, germ line development, and sexual maturation examined by qRT-PCR. cDNA from EG&K stage X, H&H stage 3, stage 6, and stage 12 (3A); blood PGCs (bPGCs), cultured PGCs (cPGCs), gonadal PGCs (gPGCs), and gonadal stromal cells (GSCs) (3B); male (3C) and female (3D) gonads on embryonic days E6.5, E8.5, E10.5, E12.5, and E14.5; and testes and ovaries from 1-day-, 4-week-, 12-week- and 25-week-old chickens were amplified with c*DNMT1*-, c*DNMT3A*-, and c*DNMT3B*-specific primers. The threshold cycle of c*DNMT* family genes were normalized with chicken glyceraldehyde-3-phosphate dehydrogenase (c*GAPDH*). Relative gene expression was calculated using the 2^−ΔΔCt^ method.

Expression of c*DNMT1*, c*DNMT3A*, and c*DNMT3B* in early embryos at stages X, 3, 6, and 12; bPGCs, cPGCs, gPGCs, and GSCs; male and female gonads at E6.5, E8.5, E10.5, E12.5, and E14.5; and testes and ovaries 1 day, 4 weeks, 12 weeks, and 25 weeks of age were examined by qRT-PCR. Relative quantification of the expression levels of each gene was normalized with c*GAPDH*. c*DNMT1* expression was detected at a low level in stage X to 12 embryos relative to c*GAPDH*. c*DNMT3A* and c*DNMT3B* expressions were initially high at stages X and 3, then decreased to a moderate level at stages 6 and 12 (Fig. 3A). When we examined the expression patterns of c*DNMT* family members in different PGC and GSC samples, c*DNMT1* and c*DNMT3A* expressions were detected in all PGC and GSC samples; however, c*DNMT1* and c*DNMT3A* expressions were slightly high in bPGCs, cPGCs, and gPGCs compared to that of GSCs. c*DNMT3B* expression was significantly high in gPGCs when compared to the other members of the c*DNMT* family. Furthermore, c*DNMT3B* expression was 11.7-fold, 6.6-fold, and 11.5-fold higher in gPGCs compared to its expression in bPGCs, cPGCs, and GSCs, respectively (Fig. 3B). Regarding the expression patterns of c*DNMT* family members during germ line development and sexual maturation, c*DNMT1* and c*DNMT3A* expressions were detected in low to moderate levels on E6.5 to 25 weeks of age in males and females. c*DNMT3B* expression was different during embryonic and post-hatch development. In males, it was significantly detected at high to moderate levels on E6.5 to E14.5. After E14.5, it decreased and was detected at a low level until 25 weeks of age. In females, c*DNMT3B* expression was significantly high at E6.5. After E8.5, it was detected at moderate to low levels until 25 weeks of age (Fig. 3C–D).

### mRNA localization of cDNMT family members during early embryonic development

**Figure 4:**
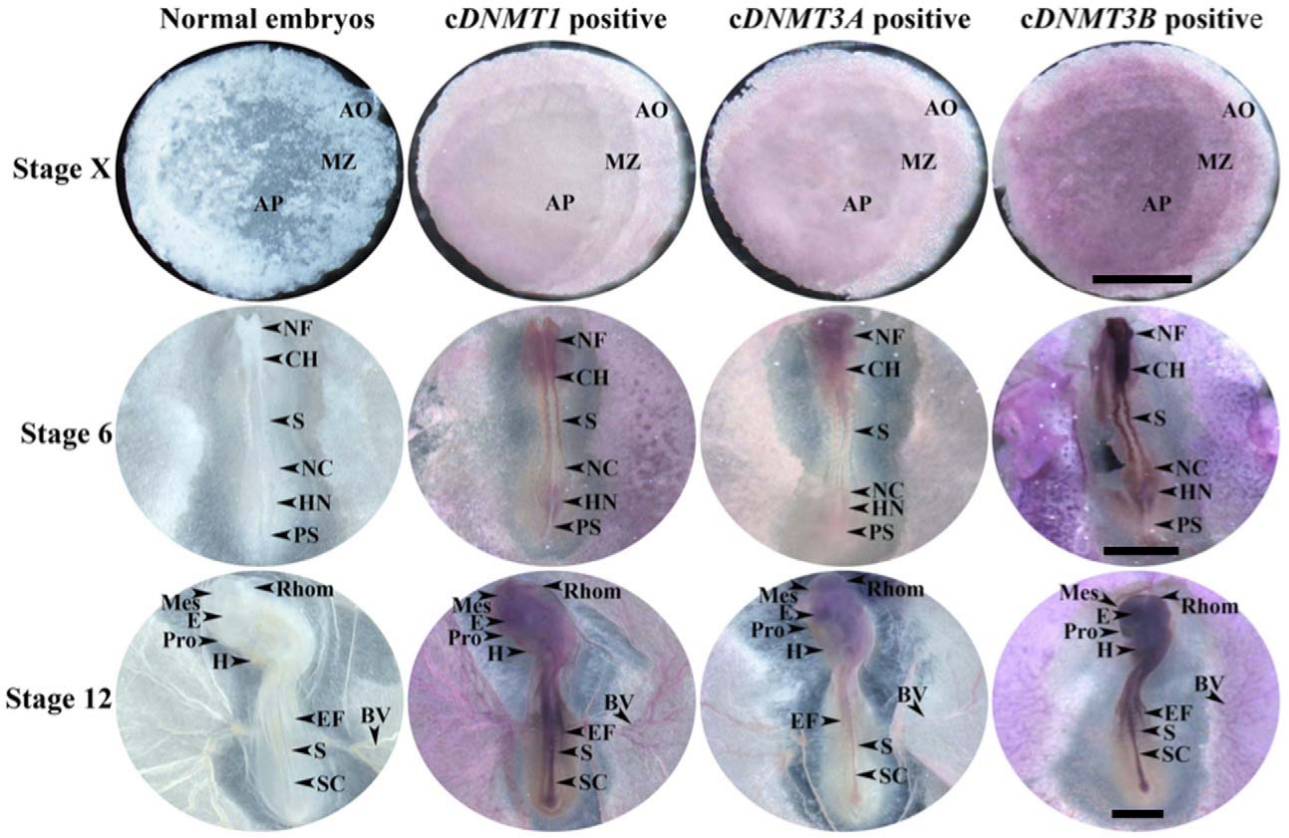
Expression patterns of c*DNMT1*, c*DNMT3A*, and c*DNMT3B* mRNA during early embryonic development. Whole-mount embryos of EG&K stage X, H&H stage 6, and stage 12 were hybridized with antisense cRNA probes against c*DNMT1*, c*DNMT3A*, and c*DNMT3B*. AP = area pellucida, MZ = marginal zone, AO = area opaca, NF = neural fold, CH = chord, S = somite, NC = notochord, HN = Hensen's node, PS = primitive streak, Pro = prosencephalon, Mes = mesencephalon, Rhom = rhombencephalon, E = eye, H = heart, EF = edge of the foregut, SC = spinal cord, BV = blood vessel. Common bar – 5 mm.

Expression patterns of c*DNMT1*, c*DNMT3A*, and c*DNMT3B* mRNA during early embryonic development at stages X, 6, and 12 were examined by whole mount *in situ* hybridization. c*DNMT1* mRNA expression was weakly detected in the area pellucida at stage X. At stage 6, c*DNMT1* mRNA expression was localized in the head fold, neural tube, and primitive streak areas. At stage 12, it was localized in all parts of the embryonic body including the head fold, optic vesicles, neural tube, ventricles, somites, and blood vessels. c*DNMT3A* mRNA expression was slightly high in the area pellucida at stage X. At stages 6 and 12, it was strongly detected in the head area compared to all other parts of the embryonic body. When compared to other members, c*DNMT3B* mRNA expression was strongly detected in the area pellucida at stage X and all parts of the developing embryos at stages 6 and 12 (Fig. 4). In addition, all c*DNMT* family members were not detected or weakly detected in the area opaca at stage X and in the yolk sac at later stages.

### DNA methylation pattern in PGCs

**Figure 5:**
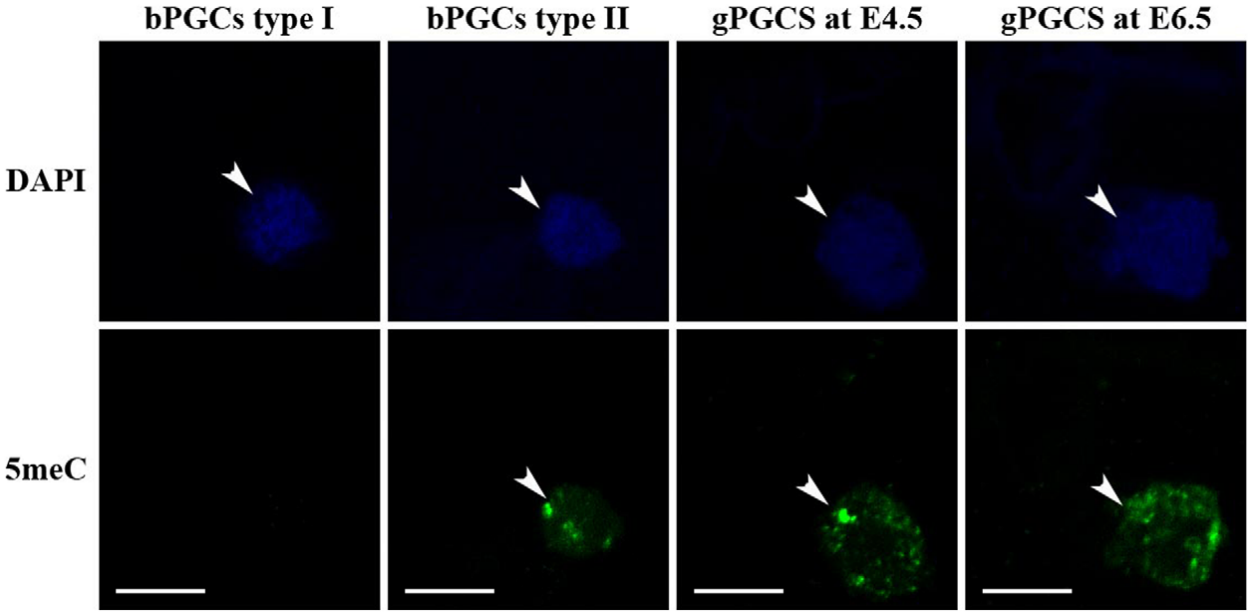
DNA methylation pattern in primordial germ cells (PGCs). Blood PGCs (bPGCs) at embryonic day E2.5 and gonadal PGCs (gPGCs) at E4.5 and E6.5 were stained with 5-methylcytosine (5 meC) and Alexa 488 dye-conjugated secondary antibody. After staining, cells were mounted with ProLongH Gold antifade reagent with 4′-6-diamidino-2-phenylindole (DAPI) and imaged with a confocal laser microscope. Arrowheads indicate DAPI and 5 meC stained PGCs. Common bar – 10 µm.

Immunocytochemical staining was performed to confirm the DNA methylation pattern in PGCs. In chicken embryos, PGCs usually occur in the circulation at around E2.0 to E3.0, and they enter differentiating germinal ridges by around E3.5 to E4.0. MACS-sorted bPGCs at E2.5, and gPGCs at E4.5 and E6.5, were subjected to 5 meC antibody staining. 5 meC expression was poorly detected in some bPGCs (designated as type I), suggesting that some bPGCs were undergoing genome-wide DNA demethylation at this stage. However, staining was detectable in many bPGCs (designated as type II), which were undergoing DNA methylation. 5 meC staining was intensive and conserved in almost all PGCs after they entered into the differentiating germinal ridges at E4.5 and settled in the gonads at E6.5 (Fig. 5).

### mRNA localization of cDNMT family members during PGCs entry into embryonic gonads

**Figure 6:**
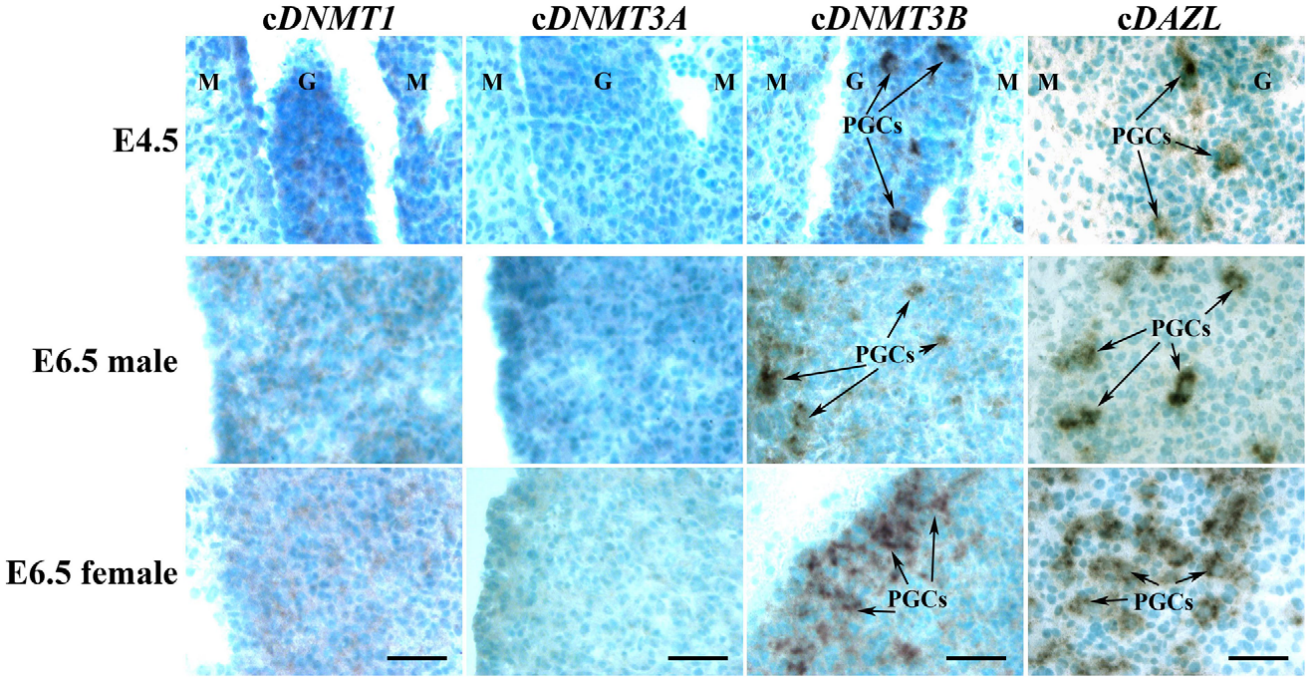
Expression patterns of c*DNMT1*, c*DNMT3A*, and c*DNMT3B* mRNA during PGCs entry into embryonic gonads compared to c*DAZL*. Transverse sections of left gonads at E4.5 and E6.5 (male and female) were hybridized with antisense cRNA probes against c*DNMT1*, c*DNMT3A*, c*DNMT3B*, and c*DAZL*. M = mesonephros, G = gonads. Arrows indicate PGCs strongly express c*DNMT3B* and c*DAZL* mRNA. Common bar – 50 µm.

Cryosections of undifferentiated and differentiated (male and female) gonads at E4.5 and E6.5, respectively, were subjected to *in situ* hybridization to examine the mRNA expression pattern of c*DNMT* family members during PGC entry into embryonic gonads. Figure 6 shows the mRNA expression pattern of c*DNMT* family members and c*DAZL* (positive control) in the left gonads of E4.5 and E6.5 embryos. c*DNMT1* mRNA expression was moderately detected in the PGCs and stromal cells at E4.5 and E6.5 gonads. c*DNMT3A* mRNA expression was detected at low levels in the PGCs, and was almost undetectable in the stromal cells. In contrast, c*DNMT3B* mRNA expression was strongly detected in the PGCs, which entered undifferentiated gonads/gonadal ridges at E4.5. After gonadal differentiation, c*DNMT3B*-positive PGCs were widespread in the male gonads. However, c*DNMT3B*-positive PGCs were detected only in the peripheral area in the female gonads. c*DNMT3B* mRNA expression was detected at very low levels in the stromal cells from E4.5 to E6.5. c*DNMT3B* mRNA expression was highly comparable to the expression pattern of c*DAZL*, a PGC and germ cell marker (Fig. 6).

### mRNA localization of cDNMT family members during germ line development and sexual maturation

**Figure 7:**
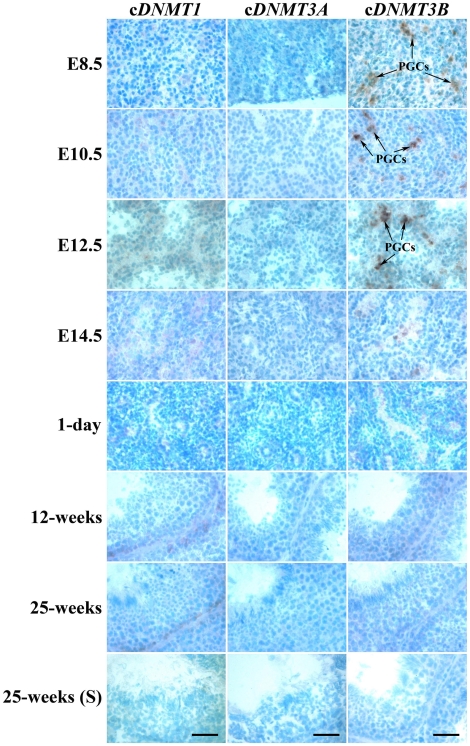
Expression patterns of c*DNMT1*, c*DNMT3A*, and c*DNMT3B* during male germ line development and sexual maturation. Cryosections of male gonads at E8.5, E10.5, E12.5, and E14.5, and testes of 1-day-, 12-week-, and 25-week-old chickens were hybridized with antisense cRNA probes against c*DNMT1*, c*DNMT3A*, and c*DNMT3B*. Testis sections at 25 weeks of age were hybridized with sense (S) cRNA probes of respective genes as negative control. Arrows indicate PGCs strongly express c*DNMT3B*. Common bar – 50 µm.

**Figure 8:**
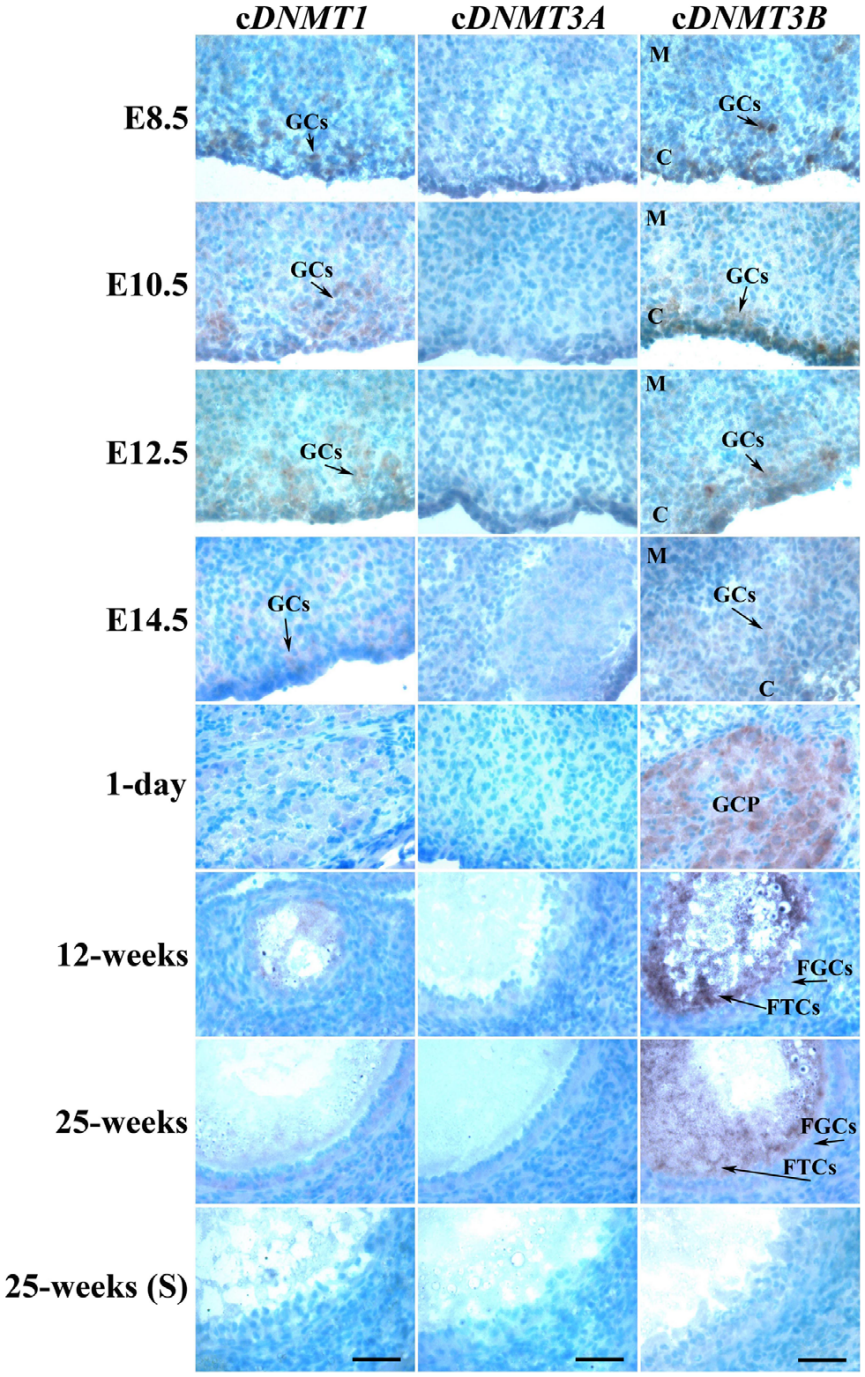
Expression patterns of c*DNMT1*, c*DNMT3A*, and c*DNMT3B* during female germ line development and sexual maturation. Cryosections of female gonads at E8.5, E10.5, E12.5, and E14.5, and ovaries of 1-day-, 12-week-, and 25-week-old chickens were hybridized with antisense cRNA probes against c*DNMT1*, c*DNMT3A*, and c*DNMT3B*. Ovary sections at 25 weeks of age were hybridized with sense (S) cRNA probes of respective genes as a negative control. C = cortex, M = medulla, GCs = germ cells, GCP = germ cell pool, FGCs = follicular granulosa cells, FTCs = follicular theca cells. Common bar – 50 µm.

Cryosections of male and female gonads at E8.5, E10.5, E12.5, and E14.5, and testes and ovaries at 1 day, 12 weeks, and 25 weeks of age were subjected to *in situ* hybridization to examine the mRNA expression patterns of c*DNMT* family members during germ line development and sexual maturation. In males, c*DNMT1* mRNA expression was detected at low levels in the PGCs (until E12.5) and prospermatogonia (on E14.5). c*DNMT1* mRNA expression was detected at a basal level in the prospermatogonia in 1-day-old testis. At 12 and 25 weeks of age, c*DNMT1* mRNA expression reappeared at a low level in the spermatogonia cells near the basement membrane. c*DNMT3A* mRNA expression was detected at a low level from E8.5 to E14.5, after which it maintained a basal level until 25 weeks of age. As expected, c*DNMT3B* mRNA expression was detected at a strong level in the PGCs (until E12.5). After PGC differentiation, it was detected at a low level in the prospermatogonia until 1 day of age and in germ line cells until testes were 25 weeks old (Fig. 7). In females, c*DNMT1* mRNA expression was detected at a moderate level in the PGCs and oogonia cells from E8.5 to E12.5. On E14.5 and 1 day of age, it was detected at a low level in the primary oocytes. From 12 weeks of age, it was detected at low levels in the secondary oocytes, maturing ova, and follicular cells. c*DNMT3A* mRNA expression was detected at a low level from E8.5 to E12.5. After E12.5, it was detected at a basal level in the ovaries. c*DNMT3B* mRNA expression was similar to c*DNMT3A* until E14.5. Interestingly, however, it was strongly detected in the primary oocytes deposited in the germ cell/oocyte pool at 1 day of age, and secondary oocytes, maturing ova, and follicular cells in 12-week-old and 25-week-old ovaries (Fig. 8).

**Figure 9:**
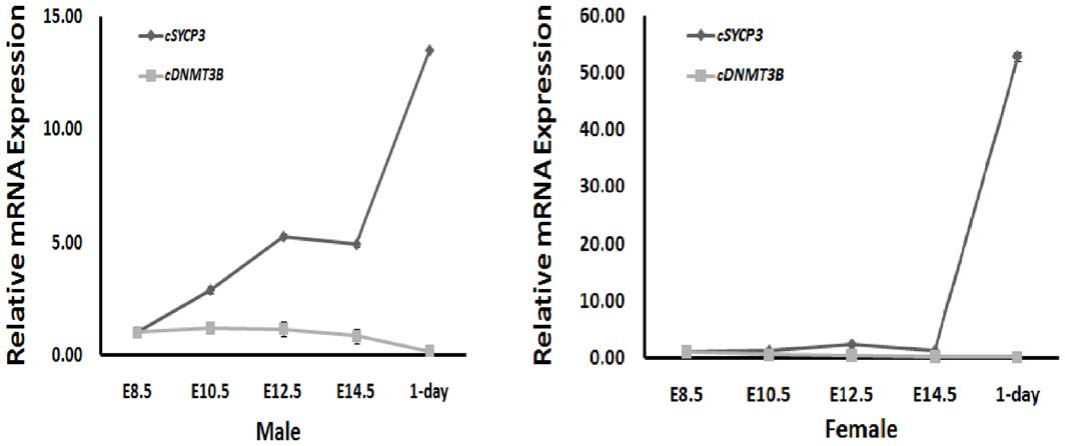
Expression of c*DNMT3B* compared with c*SYCP3* during limited points of meiotic stages examined by qRT-PCR. cDNA from male and female gonads on embryonic days E8.5, E10.5, E12.5, E14.5 and 1-day were amplified with c*DNMT3B*- and c*SYCP3*-specific primers. The threshold cycle of c*DNMT3B* and c*SYCP3* genes were normalized with chicken glyceraldehyde-3-phosphate dehydrogenase (c*GAPDH*). Relative gene expression was calculated using the 2^−ΔΔCt^ method.

In order to investigate the correlation between c*DNMT3B* expression and meiotic events, we examined the expression patterns of c*DNMT3B* with a meiosis specific gene c*SYCP3* on limited time points of meiotic stages by qRT-PCR. In males, both c*DNMT3B* and c*SYCP3* expression were detected at a low level on E8.5. After this period, c*DNMT3B* expression was continuously decreased until 1-day. However, c*SYCP3* expression was continuously increased until 1-day. In females, c*DNMT3B* expression was detected at a low level in all stages. c*SYCP3* expression was detected at al low level until E14.5. After E14.5, c*SYCP3* expression was sharply increased (Fig. 9).

### Expression patterns of miRNA and Regulation of *cDNMT3B*

**Figure 10:**
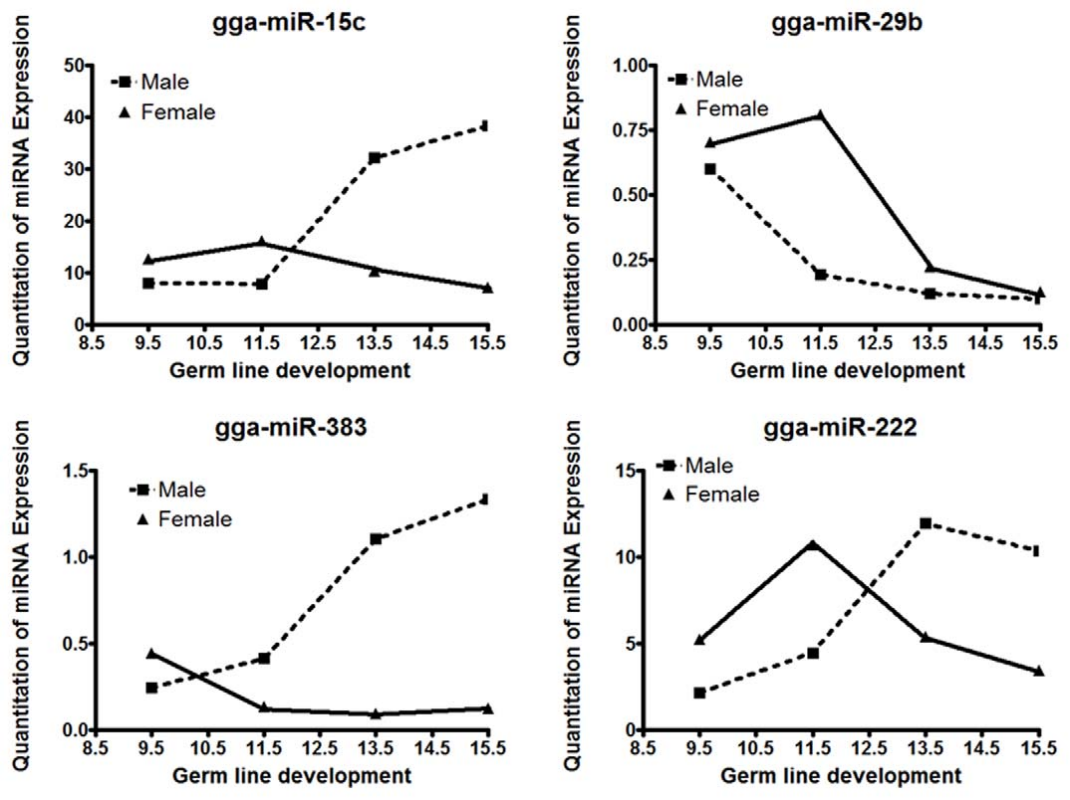
Expression of gga-miR-15c, gga-miR-29b, gga-miR-383 and gga-miR-222 during meiotic stages of germ line development examined by qRT-PCR. cDNA from male and female gonads on embryonic days E9.5, E10.5, E11.5, E12.5, E13.5, E14.5 and E15.5 were amplified with respective miRNA-specific forward primers and universal reverse primers. The threshold cycle of miRNA expression were normalized with chicken snoRNA (endogenous control).

Expression patterns of miRNAs gga-miR-15c, gga-miR-29b, gga-miR-383 and gga-miR-222 were examined during meiotic stages of germ line development by qRT-PCR. During male germ line development, miR-15c, miR-383 and miR-222 were detected at a low level on E9.5 to E11.5. After E11.5, all these miRNAs were detected at a high level. In contrast to other miRNAs, miR-29b expression was detected at a high level on E9.5, and continuously decreased until E15.5. During female germ line development, miR-15c, miR-29b and miR-222 showed similar patterns of expression. All these miRNAs were detected at a moderate level on E9.5, slightly increased until E11.5, and then continuously decreased until E15.5. miR-383 expression was detected at a moderate level on E9.5, after this period, the expression was decreased to a low level in all stages (Fig. 10).

**Figure 11:**
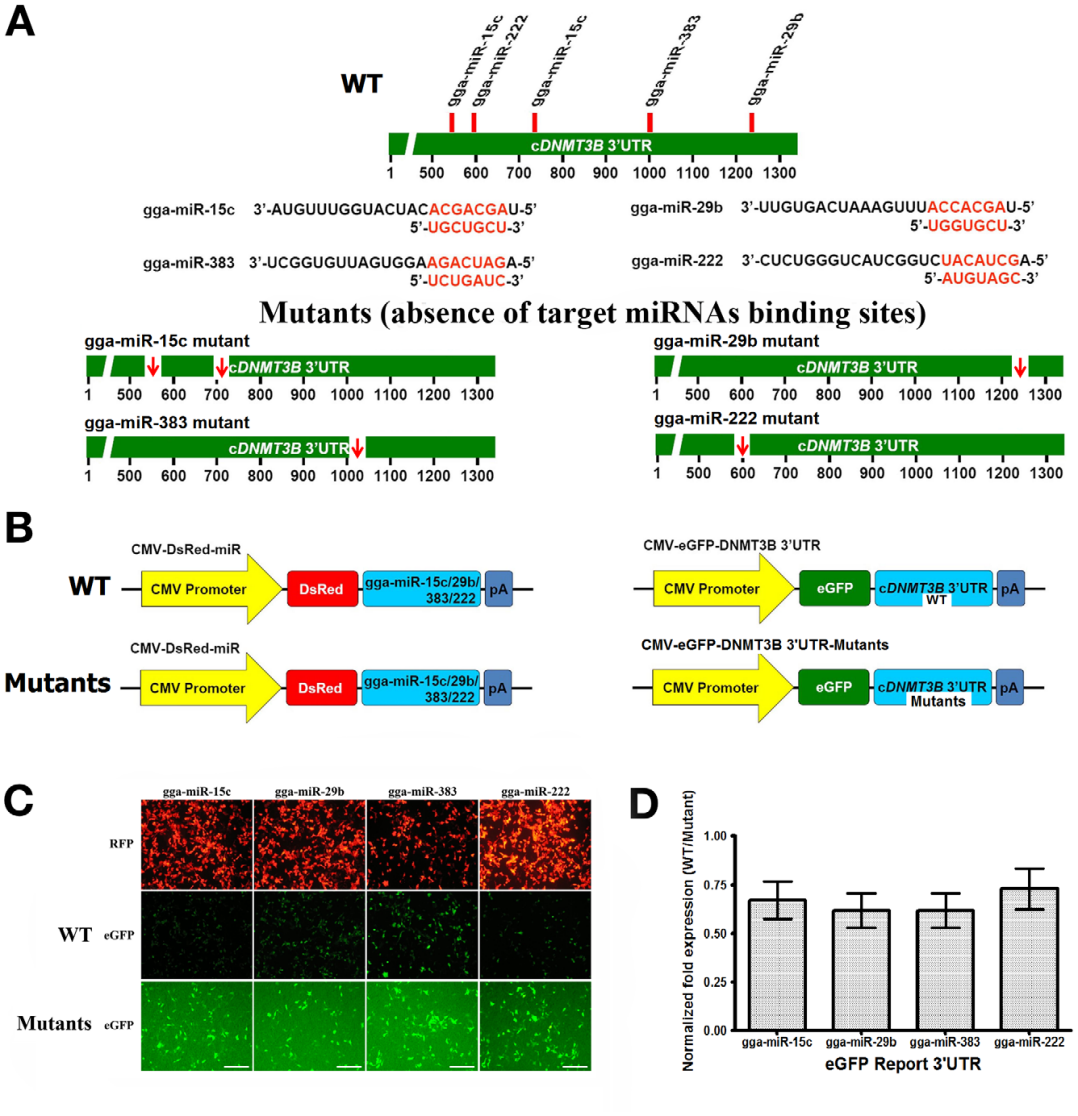
Regulation of c*DNMT3B*. (11A) The miRNA binding sites and mutants of each miRNA for c*DNMT3B* 3′UTR. (11B) Schematic diagram of constructed expression vectors for the dual fluorescent reporter assay. In the dual fluorescent reporter assay, c*DNMT3B* 3′UTR and mutants of *cDNMT3B* 3′UTR were cloned into a pcDNA3 plasmid encoding enhanced green fluorescence protein (eGFP) and c*DNMT3B* 3′UTR-specific miRNAs including gga-miR-15c, gga-miR-29b, gga-miR-383, and gga-miR-222 cloned into a pDsRed2-N1 plasmid encoding red fluorescence protein (RFP) were cotransfected into 293 FT cells. After 48 h of cotransfection, eGFP expression was examined under a confocal laser microscope (11C, Common bar – 20 µm). The inhibition rate of eGFP expression from miRNA modulation was calculated by fluorescence-activated cell sorting (FACS, 11D). The *y*-axis represents the normalized fold expression (WT vs Mutants). Error bars indicate the standard error of triplicate analysis.

To validate the downregulation of c*DNMT3B* at the post-transcription levels, the miRNAs, gga-miR-15c (target score: 74, seed location: 544 and 732), gga-miR-29b (target score: 62, seed location: 1223), gga-miR-383 (target score: 53, seed location: 996), and gga-miR-222 (target score: 50, seed location: 591) were selected for the c*DNMT3B* 3′-UTR based on an online database miRDB (Fig. 11A). Expression vectors were constructed and combined with eGFP or RFP (Fig. 11B). c*DNMT3B* 3′UTR and *cDNMT3B* 3′UTR mutants for each miRNA binding sites generated by point mutation cloned into a pcDNA3 plasmid encoding eGFP and c*DNMT3B* 3′UTR-specific miRNAs including gga-miR-15c, gga-miR-29b, gga-miR-383, and gga-miR-222 cloned into a pDsRed2-N1 plasmid, respectively, encoding RFP were cotransfected into 293 FT cells. After 48 h of cotransfection, *cDNMT3B* 3′UTR mutants encoding eGFP expression was constant as a control. c*DNMT3B* 3′UTR intensity significantly decreased in all investigated miRNAs when compared to the eGFP expression of *cDNMT3B* 3′UTR mutants (Fig. 11C). FACS analysis was performed to further examine the inhibition rate of c*DNMT3B* 3′UTR encoding eGFP expression from miRNA modulation (Fig. 11D). Compared to each mutants, c*DNMT3B* 3′UTR eGFP expression was slightly decreased by gga-miR-15c (25.82%), gga-miR-29b (30.01%), gga-miR-383 (30.0%), and gga-miR-222 (31.28%). In addition, chicken *DNMT3B* 3′UTR region is highly conserved with human and mouse *DNMT3B* 3′UTR regions. However, the c*DNMT3B* 3′UTR specific miRNAs binding sites are not conserved in human and mouse (Fig. S4).

## Discussion

The C-terminus of cDNMT1, cDNMT3A, and cDNMT3B contains the highly conserved catalytic domain DNA methylase. DNA methylase enzymes specifically methylate the C-5 carbon of cytosines to produce C5-methylcytosine. Cytosine-specific methyltransferases transfer methyl groups from S-adenosylmethionine to cytosines in CpG dinucleotides, which modulate gene expression and cell differentiation during embryogenesis [18]. The noncatalytic N-terminus of cDNMT1 is an independent domain structure that interacts with different regulatory proteins and DNA, and significantly differs from the N-terminus of cDNMT3A and cDNMT3B. The noncatalytic N-terminus of cDNMT1 consists of a DMAP1 binding domain, DNMT1-specific replication foci domain, CXXC zinc finger domain, and BAH domains, which are also conserved in mammalian DNMT1 proteins. DMAP1 is a transcriptional corepressor that binds to the N-terminal amino acids of DNMT1. DNMT1-specific replication foci domain function noncatalytically to target the proteins toward replication foci, and allow DNMT1 protein to methylate the correct residues [18]. The CXXC zinc finger domain contains eight conserved cysteine residues that bind to nonmethyl-CpG dinucleotides. The CXXC domain is found in a variety of chromatin-associated proteins [19], [20]. The BAH domain appears to act as a protein–protein interaction module specialized in gene silencing. The BAH module might play an important role by linking DNA methylation, replication, and transcriptional regulation [21]. The noncatalytic N-terminus of cDNMT3A and cDNMT3B consists of a unique domain, the Proline-Tryptophan-Tryptophan-Proline motif (PWWP domain), which binds to histone-4 methylated at lysine-20 (H4K20me). The methylation of H4K20 is involved in organizing higher-order chromatin, maintaining genome stability, and regulating cell cycle progression [22].

In mammals, many researchers have reported that *DNMTs* are expressed at high levels in undifferentiated embryonic stem cells (ESCs), embryonic carcinoma cells, germ cells, and gonads [4], [23], [24]. Our results in this investigation suggest that c*DNMT1*, c*DNMT3A*, and c*DNMT3B* are highly active in early embryos for the maintenance of methylation patterns and *de novo* methylation. During midembryonic development, c*DNMT3B* mRNA expression was strongly detected in PGCs when compared to its expression in germ cells, suggesting that c*DNMT3B* is more highly active than other members of the c*DNMT* family to ensure *de novo* methylation in PGCs. During late embryonic development and germ line development, c*DNMT* family members were weakly detected in both sexes. However, c*DNMT3B* expression was reestablished in female germ cells after hatching. DNA methylation occurs in a nonrandom manner within the genome, and the generated methylation pattern is gene- and tissue-specific. The generation of the methylation pattern requires *de novo* methylation during embryogenesis [25]. *De novo* methylation is largely suppressed in differentiated somatic cells; however, it is upregulated in germ cells and is believed to play a critical role in the establishment of genomic imprinting in the gametes [24]. c*DNMT3B* expression in follicular theca cells remains unclear. Our experiments suggest that c*DNMT3B* expression in PGCs and germ cells is higher than that of other members. On the other hand, c*DNMT1* expression in PGCs and germ cells is higher than that of c*DNMT3A*, which suggests that c*DNMT1* might be significantly involved in *de novo* methylation and interaction with c*DNMT3B*. Although its preference for hemimethylated DNA is unique among *DNMTs*, *DNMT1* also has a significant capacity for *de novo* methylation [23], [26]. In the mouse, *Dnmt1* expression is strong in PGCs, growing oocytes, and proliferating male germ cells, but is downregulated during late embryonic development, which supports our findings that genome-wide DNA methylation occurs after germ cell proliferation is arrested, when the *DNMT1* expression is downregulated [27].

miRNAs are short, noncoding RNAs that usually bind to their complementary sequences in the 3′UTR of target mRNA, resulting in gene silencing or downregulation at the post-transcriptional level [28]. Many miRNAs cloned from chicken embryos were previously reported to be ESC-specific in the mouse and human, indicating their contribution to basic cellular functions and maintenance of pluripotency [29]. Regulation of c*DNMT3B* using the c*DNMT3B* 3′UTR target miRNAs gga-miR-15c, gga-miR-29b, gga-miR-383, and gga-miR-222 is of great interest in this study. We found that c*DNMT3B* encoding eGFP expression was significantly decreased by all investigated miRNAs when compared to the control eGFP expression. To our knowledge, the expression and functions of miR-15c have not been well studied. miR-383 expression is abundant in meiotic prophase cells and primary spermatocytes in mammals [30]. miR-383 significantly interacts with growth arrest and the DNA damage-inducible gamma (*GADD45G*) gene, which inhibits cell growth and induces apoptosis. The silencing of *GADD45G* could be reversed by genetic double knockout of *DNMT1* and *DNMT3B*, indicating a direct epigenetic mechanism [31]. The expression and functions of miR-29b and miR-222 have been extensively characterized in mammals.

miR-29b directly targets *DNMT3A* and *DNMT3B*, and indirectly targets *DNMT1*, thereby leading to downregulation of genes, reduction of global DNA methylation, and re-expression of the DNA hypermethylated and silenced tumor suppressor genes [32]. miR-29b significantly regulates many collagen genes, matrix metalloproteinase 2 (*MMP2*), integrin beta1 (*ITGB1*), progranulin (*PGRN*), podoplanin (*PDPN*), and other genes related to the extracellular matrix [33], [34], [35]. Furthermore, it plays an important role during osteoblast differentiation and gonadogenesis [36], [37]. It is expressed in mouse PGCs, and its expression is upregulated in a female-specific manner when male-specific *de novo* methylation of the PGC genome occurs [37]. miR-222 induces cell growth and cell cycle progression via direct targeting of cyclin-dependent kinase inhibitors 1B and 1C (*p27* and *p57*). miR-222 significantly interacts with several target genes including: *p27* and *p57* [38], [39], [40]; phosphatase and tensin homolog (*PTEN*) [41]; estrogen receptor alpha (*ERalpha*) [42]; pro-apoptotic gene *PUMA* [43]; protein phosphatase 2A subunit B (*PPP2R2A*) [44]; and β1-syntrophin [45]. Functionally, it is involved in inflammation-mediated vascular remodeling [46], is modulated during myogenesis, and plays a role both in the progression from myoblasts to myocytes and in the achievement of the fully differentiated phenotype [47]. miR-222 also participates in ESC differentiation by regulating ESCs terminally withdrawing from the cell cycle [48].

Downregulation of *DNMT3B* causes hypomethylation in germ line cells and somatic cells [49]. Because *DNMT*s are associated with genomic imprinting, gene expression, and embryonic development, c*DNMT3B* downregulation might cause hypomethylation and downregulation of genes that are normally germ cell-specific or somatic cell-specific. We examined the expression of gga-miR-15c, gga-miR-29b, gga-miR-383 and gga-miR-222 during meiotic stages of germ line development by qRT-PCR. In correlation with c*DNMT3B* expression, gga-miR-15c, gga-miR-383 and gga-miR-222 expressions were increased after E11.5 in males. On the other hand, all four miRNAs were detected at a low level during meiotic stages in females. Earlier publications reported the downregulation of *DNMT3B* by miR-29b or miR-383 particularly in tumor cells [31], [32]. Our studies in chickens reinforce that miR-15c, miR-29b, miR-383 and miR-222 may downregulate *DNMT3B* in PGCs from female embryos after they enter meiosis. We also suggest that miR-15c, miR-383 and miR-222 may downregulate *DNMT3B* in PGCs from male embryos.

Our study highlights the bioinformatics analysis of sequence conservation and functional domains of cDNMT family proteins, and the conserved expression patterns of c*DNMT* family genes during early embryonic development, germ line development, and sexual maturation of testis and ovaries. All c*DNMT* family members were differentially expressed during early embryonic development. Of interest, expression of the *de novo* DNA methyltransferase gene c*DNMT3B* was highly detected in the early embryos and PGCs. During late germ line development and sexual maturation, c*DNMT3B* expression was reestablished in a female germ cell-specific manner. Correlation between c*DNMT3B* and miRNAs expressions during meiotic stages of germ line development suggests that gga-miR-15c, gga-miR-383 and gga-miR-222 may downregulate c*DNMT3B in vivo* in both male and female chickens. Gga-miR-29b is believed to downregulate c*DNMT3B* in a sex specific manner. Our dual fluorescent reporter assay suggests that gga-miR-29b, gga-miR-383 and gga-miR-222 may cause maximum (30.01–31.28%) downregulation of c*DNMT3B in vitro*.

## Supporting Information

Figure S1 **Sequence comparison of vertebrate DNMT1 proteins.** The protein sequences of DNMT1 from chicken, human, chimpanzee, pig, cattle, horse, rat, mouse, opossum, and zebrafish were aligned using the CLUSTAL X program and edited with the BioEdit program. Dark/light gray shaded sequences indicate amino acids identical/similar to those in chicken DNMT1, and dashes represent gaps in the sequence. Arrows indicate the positions of the DMAP1 (DNMT1-associated protein 1) binding domain, DNMT1-specific replication foci domain, CXXC zinc finger domain, BAH (bromo-adjacent homology) domains, and DNA methylase domain in the chicken sequence.
(PDF)

Figure S2 **Sequence comparison of vertebrate DNMT3A proteins.** The protein sequences of DNMT3A from chicken, human, chimpanzee, pig, cattle, horse, rat, mouse, opossum, and zebrafish were aligned using the CLUSTAL X program and edited with the BioEdit program. Dark/light gray shaded sequences indicate amino acids identical/similar to those in chicken DNMT3A, and dashes represent gaps in the sequence. Arrows indicate the positions of the PWWP domain and DNA methylase domain in the chicken sequence.
(PDF)

Figure S3 **Sequence comparison of vertebrate DNMT3B proteins.** The protein sequences of DNMT3B from chicken, human, chimpanzee, pig, cattle, horse, rat, mouse, opossum, and zebrafish were aligned using the CLUSTAL X program and edited with the BioEdit program. Dark/light gray shaded sequences indicate amino acids identical/similar to those in chicken DNMT3B, and dashes represent gaps in the sequence. Arrows indicate the positions of the PWWP domain and DNA methylase domain in the chicken sequence.
(PDF)

Figure S4 **Comparison of chicken** ***DNMT3B*** **3′UTR and** ***DNMT3B*** **3′UTR specific miRNA binding sites with human and mouse** ***DNMT3B*** **3′UTR using the CLUSTAL X program.** miR-15c, miR-29b, miR-383 and miR-222 binding sites in chicken and corresponding human and mouse sequences are shown in red colour.
(PDF)
