# Supplementary material for: Expression Patterns and miRNA Regulation of DNA Methyltransferases in Chicken Primordial Germ Cells
Source: PLoS One. 2011 May 3;6(5):e19524. doi: 10.1371/journal.pone.0019524 (PMC3086922; doi:10.1371/journal.pone.0019524)
Supplement: Figure S1 — Sequence comparison of vertebrate DNMT1 proteins. The protein sequences of DNMT1 from chicken, human, chimpanzee, pig, cattle, horse, rat, mouse, opossum, and zebrafish were aligned using the CLUSTAL X program and edited with the BioEdit program. Dark/light gray shaded sequences indicate amino acids identical/similar to those in chicken DNMT1, and dashes represent gaps in the sequence. Arrows indicate the positions of the DMAP1 (DNMT1-associated protein 1) binding domain, DNMT1-specific replication foci domain, CXXC zinc finger domain, BAH (bromo-adjacent homology) domains, and DNA methylase domain in the chicken sequence. (PDF) [file pone.0019524.s001.pdf]

DMAP1 binding domain

```
-------------------------------------------------------MPARSAPPPPALP--------PALR-RRLRDLERDEDSLSEKETL 36
-------------------------------------------------------MPARTAPARVPTLAVPAISLPDDVR-RRLKDLERD--SLTEKECV 42
MILGYRLSPFGTSPPPPRPDWGGRRRLRSSPLPIGFRAKSRGACAATAASAEASEMPARTAPARVPTLAVPAISLPDDVR-RRLKDLERD--SLTEKECV 97
-------------------------------------------------------MPARTAPARVAALASRAFSLPDDVR-RRLKDLERH--SLTEKECV 42
-------------------------------------------------------MPARTAPARVPALASRAFSLPDDVR-RRLKDLERD--SLTEKECV 42
-------------------------------------------------------MPARTAPARVPALALPAFSLPDDVRSRRLKDLERD--SLTEKECV 43
-------------------------------------------------------MPARTAPARVPALASPAGSLPDHVR-RRLKDLERD--GLTEKECV 42
-------------------------------------------------------MPARTAPARVPALASPAGSLPDHVR-RRLKDLERD--GLTEKECV 42
-------------------------------------------------------MPARTGPAPVAAPGSPALQLPEEVR-RRLKDLEKDEDILTEKECI 44
-------------------------------------------------------MPTKTS-----------LSLPEDVK-ERLQVLDEGGDSLSDEECV 33

QEKLRLTRGFLRAEVQRRLSALDADVRCRELSEERYLAKVKALLRRELAAENGDAAKLFSRASNGCAGNGEEEWERGGRGEDGAMEVEEAAASS------ 130
KEKLNLLHEFLQTEIKNQLCDLETKLRKEELSEEGYLAKVKSLLNKDLSLENGAHA--YNREVNGRLENGNQARSEARRVGMADANSPPKPLSKPRTPRR 140
KEKLNLLHEFLQTEIKNQLCDLETKLRKEELSEEGYLAKVKSLLNKDLSLENGAHA--YNREVNGRLENGNQARSEARRVGMADANSPPKPLSKPRTPRR 195
KEKLNLLHEFLQTEIKNQLCDLETKLHKEELSEEGYLAKVKSLLNKDLSLENGAHA--FSREVNGYLENGSQTSGEDRRVEMAEENKSPKPVSRLGTPRR 140
KEKLNLLHEFLRTEIKNQLCDLETKLHKEELSEEGYLAKVKSLLNKDLSLENGAHA--FSREANGCLENGSQTSGEDCRVVMAEKGKPPKPVSRLYTPRR 140
KEKLNLLHEFLQTEIKNQLCDLETKLHKEELSEEGYLAKVTSLLNKDLSSENGAPA--FSREVNGCLGNGSQARGEDRRVSMAEENKSPKLVSKLGTPRR 141
KEKLNLLHEFLQTEIKSQLCDLETKLHKEELSEEGYLAKVKTLLNKDLCLENGTLS--LTQKANGCPANGSRP---TWKAEMADSNRSPRSRPKPRGPRR 137
REKLNLLHEFLQTEIKSQLCDLETKLHKEELSEEGYLAKVKSLLNKDLSLENGTHT--LTQKANGCPANGSRP---TWRAEMADSNRSPRSRPKPRGPRR 137
KKKLSLLHEFLQAEVQHQLFDLEIKLHKEELSEEGYLAKVKFLLNKELSLENGDHT--LNQKVNGCLENGTYTSD-----EDFEKSIEEDSAVE------ 131
KEKLRLLQEFLLADTQDQLKNLEDKLKSSELSTEVYMSEVKAVLKKALGVGKEGDGVEQNGHSNGFSENGSHKDNGEQEGAMDTQD------------- 119

---------------------------SSSSSSSSSSSSSSSSSSLLPAPRARKARRSRSNGESKKSPASS--------RVTRSSGRQPTILS------ 189
SKSDGEAKRSRDPPASASQVTGIRAEPSPSPRITRKSTRQTTITSHFAKGPAKRKPQEESERAKSDESIKEEDKDQDEKRRRVTSRERVARPLP-AEEPE 239
SKSDGEAK----------------PEPSPSPRITRKSTRQTTITSHFAKGPAKRKPQEESERAKSDESIKEEDKDQDEKRRRVTSRERVARPLP-AEEPE 278
SKSDGEAKSA---------------EVSSSPRITRQTTRQTTITSHFTRGPGKRKPEEDTAKAKPDSPVEEEEKDQEEKRRKVTSRDSVAGLLP-TEEPE 224
SKSDGETKS----------------EVSSSPRITRKTTRQTTITSHFPRGPAKRKPEEEPEKVKSDDSVDEE-KDQEEKRRRVTSRERVAGLLP-AEEPG 222
SRGDGEPK----------------AEVSSSPRITRQTTRQTTITSHFARGPAKRKPEEELERAKADNSVDEE-KDQDEKRRRVTSREPVARPLP-AEEPD 223
SKSDSETMI----------------EASSSSVATRRTTRQTTITSHF-KGPAKRKPKEDSEKGNANESAAEE-RDQDKKRRVAGTESRASRAGESVEKPE 219
SKSDSDTLF----------------ETSPSSVATRRTTRQTTITAHFTKGPTKRKPKEESEEGNSAESAAEE-RDQDKKRRVVDTESGAAAA---VEKLE 217
---------------------------MEEATGSSTSLSKS------------RKPRRSKSDGETKKSSASS--------RVTRSSGKQPTIVS------ 178
----------------------------EGDAIKSPS------------APKGRGGRRSKADSEPKKSPASS--------RVTRNTGKQQTIVS------ 165

-------------------------VFSKGSTKRKS-EEVNGAVKPEVSAEKDEEE----------------------------------EEELEEKEQD 229
RAKSGTRTEKEEERDEKEEKR-LRSQTKEPTPKQKLKEEPDREARAGVQADEDEDG-DEKDEKKHRSQPKDLAAKRRPEEKEPEKVNPQISDEKDEDEKE 337
RAKSGTRTEKEEERDEKEEKR-LRSQTKEPTPKQKLKEEPDREARAGVQADEDEDG-DEKDEKKHRSQPKDLAAKRRPEEKEPEKVNPQISDEKDEDEKE 376
RVRPGTHMEE----DDKEEKR-LRSQTKELTPKQKIKEELDRSTRPGGAQPGTNE--EDKDEKRHRSQPKGLAGKRRPEEKEPERIKPQVSDEKDEDEKE 317
RVRPGTHMEEEG-RDDKEEKR-LRSQTKEPTPKHKAKEEPDRDVRPGGAQAEMNEG-EDKDEKRHRSQPKDLASKRRPEEKEPERVKPQVSDEKDEDEKE 319
RVRPGTHTEEEEEREDREEKR-VRSQTKEPTPKQKPKEEPDREARPGAQAEVDEGG-EERDEKRHRSQPKDLAAKRRPEEKEPERVKPQVSDEKDEDEKE 321
RVRPGTQLCQEEQGEQEDDRR-PRRQTRELASRRKSREDPDREARPGTHLDVDDDDEKDKRSSRPRSQPRDLATKRRPKE-EVEQITPEPPEGKDEDERE 317
EVTAGTQLGPEEPCEQEDDNRSLRRHTRELSLRRKSKEDPDREARPETHLDEDEDGKKDKRSSRPRSQPRDPAAKRRPKEAEPEQVAPETPEDRDEDERE 317
-------------------------LFSKGSSKRKS-EGATGEIKQEMNVEK--------------------------------------EDDLEEKEQG 214
--------------------------MFSRVPKRKSDELNGEPANGDTEIKT--------------------------------------EETITEEVRE 201

EKRIKIETKEG--------SEIKDEITQVKTSTPA-KTTPPKCVDCRQYLDDPDLKFFQGDPDDALEEPEMLTDERLSIFDANEDGFESYEDLPQHKVTS 320
EKRRKTTPKEP----------TEKKMARAKTVMNS-KTHPPKCIQCGQYLDDPDLKYGQ-HPPDAVDEPQMLTNEKLSIFDANESGFESYEALPQHKLTC 425
EKRRKTTPKEP----------TEKKMARAKTVVNS-KTHPPKCIQCGQYLDDPDLKYGQ-HPPDAVDEPQMLTNEKLSIFDANESGFESYEALPQHKLTC 464
EKRRRTTYKEP----------TEKKLARTKTAVVSTKADPLKCVQCGQYLDDAELKYEQ-HPPDAVEEIQLLTNERLSIFDANESGFESYEALLQHKLTG 406
EKRRRTTYREL----------TEKKMTRTKIAVVS-KTNPPKCTECLQYLDDPELRYEQ-HPPDAVEEIQILTNERLSIFDANESGFESYEDLPQHKLTC 407
EKRRKTTSKEP----------MEKKMARTKTVLSS-KIHPPRCVQCGQYLDDAELKYEQ-HAPDAVDEIQLLTNERLSIFDANESGFESYEALPQHKLTC 409
EKRRKTTRKKPEPLSIPVQSRVERKASQGKASAIP-KLNPPQCPECGQYLDDPDLKYQQ-HPVDAVDEPQMLTNEALSVFDSNSSWFETYDSSPMHKFTF 415
EKRRKTTRKKLESHTVPVQSRSERKAAQSKS-VIP-KINSPKCPECGQHLDDPNLKYQQ-HPEDAVDEPQMLTSEKLSIYDSTSTWFDTYEDSPMHRFTS 414
EKKMKFEVKEG--------SEIK-EVVQGKAVPPV-KSTPPKCMDCRQYLDDPDLKFFQGDPDGALDEPEMLTDERLSIFDANEDGFESYDDLPQHRVTS 304
EKRLKTEDEKP-----------EAENAANLKPVSTAKTPPPKCPDCRQYLDDSDLKFFQGDPDDALDEPEMLTDERLSLFDSNEDGFESYEDLPQHKITN 290
```

Replication foci domain

```
FSVYDKRGHLCPFDTGLIERNIELYFSGAVKPIYDDNPCLDGGVRAKKLGPINAWWITGFDGGEKALIGFTTAFADYILMEPSEEYAPIFALMQEKIYMS 420
FSVYCKHGHLCPIDTGLIEKNIELFFSGSAKPIYDDDPSLEGGVNGKNLGPINEWWITGFDGGEKALIGFSTSFAEYILMDPSPEYAPIFGLMQEKIYIS 525
FSVYCKHGHLCPIDTGLIEKNIELFFSGSAKPIYDDDPSLEGGVNGKNLGPINEWWITGFDGGEKALIGFSTSFAEYILMDPSPEYAPIFGLMQEKIYIS 564
FSVYCKRGHLCPIDTGLIEKDVELFFSGSAKPIYEDDPSLEGGVNGKNLGPINEWWITGFDGGEKALIGFSTSFAEYILMDPNPEYAPLFSVMQEKIYIS 506
FSVYCKRGHLCPIDTGLIEKDVELLFSGSAKPIYEDDPSPEGGINGKNFGPINEWWIAGFDGGEKALLGFSTSFAEYILMDPSPEYAPLFSVMQEKIYIS 507
FSVYCKRGHLCPIDTGLIEKNVELFFSGSAKPIYDDDPSLEGGVNGKDLGPINEWWITGFDGGEKALIGFSTSFAEYILMDPNPEYAPMFSVMQEKIYIS 509
FSVYCSRGHLCPVDTGLIEKNVELYFSGVAKAIHEENPSVEGGVNGKNLGPINQWWISGFDGGEKALIGFSTAFAEYFLMEPSPEYAPIFGLMQEKIYIS 515
FSVYCSRGHLCPVDTGLIEKNVELYFSGCAKAIHDENPSMEGGINGKNLGPINQWWLSGFDGGEKVLIGFSTAFAEYILMEPSKEYEPIFGLMQEKIYIS 514
FSVYDKKGHLCPFDTGLIEKNIELYFSGTVKPIYDDNPCLDGGVRAKKLGPINAWWITGFDGGEKALIGFTTAFADYILMAPSEEYAPIFALMQEKIYMS 404
FSVYDKRGHLCPFDSGLIEKNVELYFSCAVKPIYDDNPCMDGGVPAKKLGPINAWWITGFDGGEKALIGFTTAFADYILMDPSEEYSAIFALMQEKICMS 390

KIVVEFLQNNRDVSYEDLLNKIETTVPPVGLNFNRFTEDSLLRHAQFVVEQVESYDEAGDSDEPPVLITPCMRDLIKLAGVTLGKRRAV-RRQAIRHPTR 519
KIVVEFLQSNSDSTYEDLINKIETTVPPSGLNLNRFTEDSLLRHAQFVVEQVESYDEAGDSDEQPIFLTPCMRDLIKLAGVTLGQRRAQARRQTIRHSTR 625
KIVVEFLQSNSDSTYEDLINKIETTVPPSGLNLNRFTEDSLLRHAQFVVEQVESYDEAGDSDEQPIFLTPCMRDLIKLAGVTLGQRRAQARRQTIRHSTR 664
KIVVEFLQNNPDSTYEDLINKIETTVPPSVLNLNRFTEDSLLRHAQFVVEQVESYDQAGDSDEQPIFLTPCMRDLIKLAGVTLGKRRAE-RRRTIGHSTK 605
KIVVEFLQSNPDSTYEDLINKIETTVPPCMLNLNRFTEDSLLRHAQFVVEQVESYDRAGDSDEQPIFLSPCMRDLIKLAGVTLGKRRAE-RRQTIRQPAK 606
```

```
KIVVEFLQSNPDSTYEDLINKIETTVPPSVLNLNRFTEDSLLRHAQFVVEQVESYDEAGDSDEQPIFLTPCMRDLIKLAGVTLGKRRAE-RRQTIGHSAK 608
KIVVEFLQSNPDAVYEDLINKIETTVPPSAINVNRFTEDSLLRHAQFVVSQVESYDDAKDDDETPIFLSPCMRSLIHLAGVSLGQRRAT--RRTVINSAK 613
KIVVEFLQNNPDAVYEDLINKIETTVPPSTINVNRFTEDSLLRHAQFVVSQVESYDEAKDDDETPIFLSPCMRALIHLAGVSLGQRRAT--RR-VMGATK 611
KIVVEFLQNNPDVSYEDLLNKIETTVPPAGLNFNRFTEDSLLRHAQFVVEQVESYDEAGDSDEQPVIITPCMRDLIKLAGVTLGKRRAA-RRQAIRHPTK 503
KIVVEFLQKNQDATYEDLLNKIETTVPPAGLNFNRFTEDTLLRHAQFVVEQVESYDEAGDSDEQPIIITPCMRDLIKLAGVTLGKRRAA-RRQAVRHPTK 489

                                            → CXXC zinc finger domain ←
ID-KDKGPTKATTTKLVYLIFDTFFSEQIEKDEREDDKENAMKRRRCGVCEVCQQPECGKCKACQNMVKFGGSGRSKQACLQRRCPNLAVREADEDEEVD 618
E--KDRGPTKATTTKLVYQIFDTFFAEQIEKDDRED-KENAFKRRRCGVCEVCQQPECGKCKACKDMVKFGGSGRSKQACQERRCPNMAMKEADDDEEVD 722
E--KDRGPTKATTTKLVYQIFDTFFAEQIEKDDRED-KENAFKRRRCGVCEVCQQPECGKCKACKDMVKFGGSGRSKQACQERRCPNMAMKEADDDEEVD 761
E--KDKGPTKATTTKLVYQIFDTFFAEQIEKDDKED-KENAFKRRRCGVCEVCQQPECGKCKACKDMVKFGGSGRSKQACQERRCPNMAMKEADDDEEVD 702
E--KDKGPTKATTTKLVYQIFDTFFAEQIEKDDKED-KENAFKRRRCGVCEICQQPECGKCKACKDMVKFGGSGRSKQACQKRRCPNMAMKEADDDEEVD 703
E--KDKGPTKATTTKLVYQIFDTFFAEQIEKDDRED-KENAFKRRRCGVCEVCQQPECGKCKACKDMVKFGGSGRSKQACQERRCPNMAMKEADDDEEVD 705
V--KRKGPTKATTTKLVYQIFDTFFSEQIEKDDKED-KENTMKRRRCGVCEVCQQPECGKCKACKDMVKFGGTGRSKQACLKRRCPNLAVKEADEDEEAD 710
E--KDKAPTKATTTKLVYQIFDTFFSEQIEKYDKED-KENAMKRRRCGVCEVCQQPECGKCKACKDMVKFGGTGRSKQACLKRRCPNLAVKEADDDEEAD 708
ID-KDKGPTKATTTKLVYLIFDTFFSEQIEKNEREDDKENAMKRRRCGVCEVCQQPECGKCKACQDMVKFGGSGRSKQACLQRRCPNLAVKEADEDEEVD 602
IEKDNKGPTKATTTKLVHLIFDTFFSDQIDQNNKDG----GVKRQRCGVCEVCQAPDCGKCSACKDMIKFGGSGRSKQACQKRRCPNLAVKEAEDDENMD 585

                                                  → BAH domain
DN-IPEMPSPKKMLQGRKKKQNKSRISWVGEPIKSDGKKDFYQRVCIDSETLEVGDCVSVSPDDPTKPLYLARVTAMWEDSSG-QMFHAHWFCPGSDTVL 716
DN-IPEMPSPKKMHQGKKKKQNKNRISWVGEAVKTDGKKSYYKKVCIDAETLEVGDCVSVIPDDSSKPLYLARVTALWEDSSNGQMFHAHWFCAGTDTVL 821
DN-IPEMPSPKKMHQGKKKKQNKNRISWVGEAVKTDGKKSYYKKVCIDAETLEVGDCVSVIPDDSSKPLYLARVTALWEDSSNGQMFHAHWFCAGTDTVL 860
DN-IPEMPSPKKMHQGKKKKQNKDRISWIGEAVKTDGKKIYYKKVCIDSETLEVGDCVSVIPDDSSKPLYLARVTALWEDSSNGQMFHAHWFCAGIDTVL 801
DN-IPEMPSPKKMHQGKKKKQNKNRISWVGDAVKTDGKKSYYKKVCIDSETLEVGDCVSVIPDDSSKPLYLARVTALWEDSSNGQMFHAHWFCAGTDTVL 802
DN-IPEMPSPKKMHQGKKKKQNKNRITWVGDAVKTDGKKSFYKQVCIDSETLEVGDCVSVIPDDSSKPLYLARVTALWEDSSNGQMFHAHWFCAGTDTVL 804
DD-IPELPSPKKLHQGKKKKQNKDRISWLGEPVKIEENRTYYWKVSIDEETLEVGDCVSVIPDDPSKPLYLARVTALWEDKNG-QMFHAHWFCAGTDTVL 808
DD-VSEMPSPKKLHQGKKKKQNKDRISWLGQPMKIEENRTYYQKVSIDEEMLEVGDCVSVIPDDSSKPLYLARVTALWEDKNGQMMFHAHWFCAGTDTVL 807
DN-IPEMPSPKKMLQGRKKKQNKTRISWFGAPIKSDGKKDYYQKVCIDSEILEVGDCVSVSPDDPTKPLYLARITALWEDSSG-QMFHAHWFCAGIDTVL 700
EEDVLPVKDTKKMSQTKKKKQTKNKISWVGEPLKTEGKKEYYMKVRVENEVLEVGDCVSVSPDDPSHPLYLARITALWDDGEK--MFHAHWFCRGTDTVL 683

                                                                   ←
GATSDPLELFLVDECEDMQLSYIHGKVNVIYKPPSENWAMEGGLDMEIKMV-EDDGRTYFYQMWYDQEYARFETPPRAQPMEDNKYKFCLSCARLDEVRH 815
GATSDPLELFLVDECEDMQLSYIHSKVKVIYKAPSENWAMEGGMDPESLLE-GDDGKTYFYQLWYDQDYARFESPPKTQPTEDNKFKFCVSCARLAEMRQ 920
GATSDPLELFLVDECEDMQLSYIHSKVKVIYKAPSENWAMEGGMDPESLLE-GDDGKTYFYQLWYDQDYARFESPPKTQPTEDNKFKFCVSCARLAEMRQ 959
GATSDPLELFLVDEGEDMQLSYIHSKVKVIYKPPSENWALEGGMDPEALMS-KDDGKTYFYQLWYDQEYARFESPPKTQPTEDNKFKFCVSCARLAEMRQ 900
GATSDPLELFLVDECEDMQLSYIHSKVQVIYKAPSENWAMEGGVDPEALMS-EDDGKTYFYQLWYDQDYARFESPPKTQPTEDNKYKFCASCARLAEMRQ 901
GATSDPLELFLVDECEDMQLSYIHSKVKVVYKAPSENWALEGGMDPEALMA-EDDGKTYFYQLWYDQDYARFESPPKTQPTEDNKYKFCASCARLAEMRQ 903
GATSDPLELFLVGECENMQLSYIHSKVKVIYRGPSPNWAMEGGMDPEAMLPGAEDGKTYFYQFWYSQDYARFESPPKTQPTEDNKHKFCLSCIRLAELRQ 908
GATSDPLELFLVGECENMQLSYIHSKVKVIYKAPSENWAMEGGTDPETTLPGAEDGKTYFFQLWYNQEYARFESPPKTQPTEDNKHKFCLSCIRLAELRQ 907
GATSDPLELFLVDECEDMQLSYIHGKVNVIYKAPSENWSMEGGLDMEIKMV-EDDGRTYFYQMWYDQDYARFESPPKIQSTEENKYKFCISCARLAEMRQ 799
GESSDPLELFLVDECEDMQLSFIHGKVNVFYKAPSENWYMEGGMDEDIKVI-DDDGESFFYQLHYEGECARFETPPKVTPSEDCKYKFCASCTRNKEREA 782

                        → BAH domain
KEIPKVAEPLDE--GDGKMFYAMATKNGVQYRVGDSVYLLPEAFSFSMKPASPAKR-PKKEAVDEDLYPEHYRKYSEYIKGSNLDAPDPYRVGRIKEIFC 912
KEIPRVLEQLED--LDSRVLYYSATKNGILYRVGDGVYLPPEAFTFNIKLSSPVKR-PRKEPVDEDLYPEHYRKYSDYIKGSNLDAPEPYRIGRIKEIFC 1017
KEIPRVLEQLED--LDSRVLYYSATKNGILYRVGDGVYLPPEAFTFNIKLSSPVKR-PRKEPVDEDLYPEHYRKYSDYIKGSNLDAPEPYRIGRIKEIFC 1056
KEVPRVMEQLED--LDGRVLYSSATKNRIQYRVGDGVYLPPEAFTFNIKLSSPVKG-PRKEPVDEDLYPEHYRKYSDYIKGSNLDAPDAYRIGRIKEIFC 997
KEIPRVVEQLQD--LEGRVLYSLATKNGVQYRVGDGVYLPPEAFTFNIKLSSPVKR-PRKEPVDEALYPEHYRKYSDYIKGSNLDAPEPYRIGRIKEIFC 998
KEIPRVLEQLED--LDGRVLYSSATKDGVQYRVGDGVYLLPEAFTFNIKLSSPVKR-PRKEPVDEDLYPEHYRKYSDYIKGSNLDAPEPYRIGRIKEIFC 1000
KEMPKVLEQLEE--VDGRVYCSSITKNGVVYRLGDSVYLPPEAFTFNIKMASPMKR-SKRDPVNENLYPEHYRKYSDYIKGSNLDAPEPYRIGRIKEIHC 1005
KEMPKVLEQIEE--VDGRVYCSSITKNGVVYRLGDSVYLPPEAFTFNIKVASPVKR-PKKDPVNETLYPEHYRKYSDYIKGSNLDAPEPYRIGRIKEIHC 1004
KEIPKVTEPLEE--LDHKVLYGLAMKNGVQYRTGDGVFFLPEAFGFNMKLCSPTKR-SKESVDEELYPEHYRKYSEYIKGSNQDAPDPYRIGRIKEIFC 896
ESVPHAYEPLEDEESDSKVFYGLVNYKGEQYKVGDSVYLPPEAFNFVVKAASPVKRSHRKDDVDEDLYPEYYRKSSDYIKGSNLDAPQPFRIGRIKEIFC 882

                                                                                    ←
HIRTNGKPNEADIKLRIWKFYRPENTHKSMKATYHADINLLYWSDEETTVDFCAVQGRCTVVYGEDLTESIQDYSAGGLDRFYFLEAYNAKTKSFEDPPN 1012
PKKSNGRPNETDIKIRVNKFYRPENTHKSTPASYHADINLLYWSDEEAVVDFKAVQGRCTVEYGEDLPECVQVYSMGGPNRFYFLEAYNAKSKSFEDPPN 1117
PKKSNGRPNETDIKIRVNKFYRPENTHKSTPASYHADINLLYWSDEEAVVDFKAVQGRCTVEYGEDLPECVQVYSMGGPNRFYFLEAYNAKSKSFEDPPN 1156
TKKSNGKPNETDIKIRLNKFYRPENTHKSTPASYHADINLLYWSDEEAVVDFKSVQGRCTVEYGEDLPECLQDFSAGGPDRFYFLEAYNAKSKSFEDPPN 1097
SKKSNGRPNETDIKIRVNKFYRPENTHKSTPASYHADINLLYWSDEEAVVDFKAVQGRCTVEYGEDLPQCLQDFSAGGPDRFYFLEAYNAKSKSFEDPPN 1098
IKKSNGRPNETDIKIRLNKFYRPENTHKSTPATYHADINLLYWSDEEAVVDFKAVQGRCTVEYGSEDLPECLQAFSAGGPDRFYFLEAYNAKSKSFEDPPN 1100
GKKKGGKVNEADIKIRLYKFYRPENTHKSIQATYHADINLLYWSDEEAVVDFSDVQGRCTVEYGEDLLESIQDYSQGGPDRFYFLEAYNSKTKSFEDPPN 1105
GKKKG-KVNEADIKLRLYKFYRPENTHRSYNGSYHTDINMLYWSDEEAVVNFSDVQGRCTVEYGEDLLESIQDYSQGGPDRFYFLEAYNSKTKNFEDPPN 1103
SKRSNGKPNEADIKLRVNKFYRPENTHKSMKASHHADINLLYWSDEEAIVDFKAVQGRCVIEYGEDLTECIQDYSAGGSDRFYFLEAYNAKTKSFEDPPN 996
NKRSNGKPDTTEIKLRLYKFYRPENTHKGPKGAYHSDINQLYWSDEEATVSMTEVLTRCRVEYAEDLVESVQDYSNKGPDRFYFLEAYNAKTKSFEDPPN 982

                                         → DNA Methylase domain
HARSSGNKGKGKGKGKGKGKGKSSTTCEQSEPEPTELKLPKLRTLDVFSGCGGLSEGFHQAGVSETLWAIEMWEPAAQAFRLNNPGTTVFTEDCNVLLKL 1112
HARSPGNKGKGKGKGKGKPKSQA---CEPSEPE-IEIKLPKLRTLDVFSGCGGLSEGFHQAGISDTLWAIEMWDPAAQAFRLNNPGSTVFTEDCNILLKL 1213
HARSPGNKGKGKGKGKGKPKSQA---CEPSEPE-IEIKLPKLRTLDVFSGCGGLSEGFHQAGISDTLWAIEMWDPAAQAFRLNNPGSTVFTEDCNILLKL 1252
HARSPGNKGKGKGKGKSRTKSQT---CEPSELE-TEIKLPKLRTLDVFSGCGGLSEGFHQAGISETLWAIEMWDPAAHAFRLNNPGSTVFTEDCNVLLKL 1193
HARSTGNKGKGKGKGKNRTKSQT---CEPSELE-TEIKLPKLRTLDVFSGCGGLSEGFHQAGISETLWAIEMWDPAAQAFRFNNPGSTVFTKDCNVLVKL 1194
HARSPGNKGKGKGKGKGKAKSQV---CEPSEPK-AEIKLPKLRTLDVFSGCGGLSEGFHQAGISETLWAIEMWDPAAQAFRLNNPGSTVFTEDCNVLLKL 1196
HARSPGNKGKGKGKGKGKGKPQV---SEPKEPE-AAIKLPKLRTLDVFSGCGGLTEGFHQAGISETLWAIEMWEPAAQAFRLNNPGTTVFTEDCNVLLKL 1201
HARSPGNKGKGKGKGKGKGKHQV---SEPKEPE-AAIKLPKLRTLDVFSGCGGLSEGFHQAGISETLWAIEMWDPAAQAFRLNNPGTTVFTEDCNVLLKL 1199
HARSPGNKGKGKGKGKGKSRGKS-IAVEPSEHETIEVKPPKLRTLDVFSGCGGLSEGFHQAGISETLWAIEMWEPAAQAFRLNNPGTTVFTEDCNVLLKL 1095
HARSAVNKGKGKGKGKGKGKGKQ--RSRTTGSGAQEPVVPKLRTLDVFSGCGGLSEGFHQAGISETHWAIEMWDPAAQAFRLNNPGTTVFTEDCNVLLKL 1080
```

```
VMSGEKTNSLGQKLPQKGDVEMLCGGPPCQGFSGMNRFNSRTYSKFKNSLVVSFLSYCDYYRPRFFLLENVRNFVSFKRSMVLKLTLRCLVRMGYQCTFG 1212
VMAGETTNSRGQRLPQKGDVEMLCGGPPCQGFSGMNRFNSRTYSKFKNSLVVSFLSYCDYYRPRFFLLENVRNFVSFKRSMVLKLTLRCLVRMGYQCTFG 1313
VMAGETTNSRGQRLPQKGDVEMLCGGPPCQGFSGMNRFNSRTYSKFKNSLVVSFLSYCDYYRPRFFLLENVRNFVSFKRSMVLKLTLRCLVRMGYQCTFG 1352
VMAGEVTNSRGQKLPQKGDVEMLCGGPPCQGFSGMNRFNSRTYSKFKNSLVVSFLSYCDYYRPRYFLLENVRNFVSFKRSMVLKLTLRCLVRMGYQCTFG 1293
VMAGEVTNSRGQKLLQKGDVEMLCGGPPCQGFSGMNRFNSRTYSKFKNSLVVSFLSYCDYYRPRYFLLENVRNFVSFKRSMVLKLTLRCLVRRGYQCTFG 1294
VMAGEVTNSRGQKLPQKGDVEMLCGGPPCQGFSGMNRFNSRTYSKFKNSLVVSFLSYCDYYRPRYFLLENVRNFVSFKRSMVLKLTLRCLVRMGYQCTFG 1296
VMAGEVTNSLGQRLPQKGDVEMLCGGPPCQGFSGMNRFNSRTYSKFKNSLVVSFLSYCDYYRPRFFLLENVRNFVSFRRSMVLKLTLRCLVRMGYQCTFG 1301
VMAGEVTNSLGQRLPQKGDVEMLCGGPPCQGFSGMNRFNSRTYSKFKNSLVVSFLSYCDYYRPRFFLLENVRNFVSYRRSMVLKLTLRCLVRMGYQCTFG 1299
VMSGEKTNSLGQKLPQKGDVEMLCGGPPCQGFSGMNRFNSRTYSKFKNSLVVSFLSYCDYYRPRFFLLENVRNFVSFKRSMVLKLTLRCLVRMGYQCTFG 1195
VMSGEKTNSLGQKLPQKGDVEMLCGGPPCQGFSGMNRFNSRTYSKFKNSLVVSYLSYCDYYRPKFFLLENVRNFVSFKRSMVLKLTLRCLVRMGYQCTFG 1180

VLQAGQYGVAQTRRRAIVLAAAPGEKLPMFPEPLHVFAPRACQLSVVVDDKKFVSNITRTYSGPFRTITVRDTMSDLPEIRNGASALEISYNGEPQSWFQ 1312
VLQAGQYGVAQTRRRAIILAAAPGEKLPLFPEPLHVFAPRACQLSVVVDDKKFVSNITRLSSGPFRTITVRDTMSDLPEVRNGASALEISYNGEPQSWFQ 1413
VLQAGQYGVAQTRRRAIILAAAPGEKLPLFPEPLHVFAPRACQLSVVVDDKKFVSNITRLSSGPFRTITVRDTMSDLPEVRNGASALEISYNGEPQSWFQ 1452
VLQAGQYGVAQTRRRAIILAAAPGEQLPLFPEPL--FAPRACQLSVVVDDKKFVSNITRLSSGPFRTITVRDTMSDLPEIRNGASAQDISYNGEPQSWFQ 1391
VLQAGQYGVAQTRRRAIILAAAPGEPLPLFPEPLHVFAPRACQLSVVVDDKKFVSNITRLSSGPFRTITVRDTMSDLPEIRNGASALEISYNREPQSWFQ 1394
VLQAGQYGVAQTRRRAIILAAAPGEKLPLFPEPLHVFAPRACQLSVVVDDRKFVSNITRLSSGPFRTITVRDTMSDLPEVRNGASALEISYNGEPQSWFQ 1396
VLQAGQYGVAQTRRRAIILAAAPGEKLPLFPEPLHVFAPRACQLSVVVDDKKFVSNITRLSSGPFRTITVRDTMSDLPEIQNGASAPEISYNGEPQSWFQ 1401
VLQAGQYGVAQTRRRAIILAAAPGEKLPLFPEPLHVFAPRACQLSVVVDDKKFVSNITRLSSGPFRTITVRDTMSDLPEIQNGASNSEIPYNGEPLSWFQ 1399
VLQAGQYGVAQTRRRAIILAAAPGEKLPMFPEPLHVFAPRACQLSVVVDDKKFVSNITRMNSAPFRTITVRDTMSDLPEIRNGASALEISYNGEPQSWFQ 1295
VLQAGQYGVAQTRRRAIILAAAPGEKLPRYPEPLHVFAPRACSLSVAVDEKKYVSNVTRGNGGIYRTITVRDTMSDLPEIRNGAAALEISYNGEPQSWFQ 1280

RQIRGSQYQPILRDHICKDMSALVAARMRHIPLAPGSDWRDLPNIEVRLSDGTSTRKLRYTHHEKKNGRSSSGALRGVCSCAE-GKPCDPADRQFNTLIP 1411
RQLRGAQYQPILRDHICKDMSALVAARMRHIPLAPGSDWRDLPNIEVRLSDGTMARKLRYTHHDRKNGRSSSGALRGVCSCVEAGKACDPAARQFNTLIP 1513
RQLRGAQYQPILRDHICKVMAPRQSG---------------------------------------SSSRPSPAASWEQSACPRLG--------------- 1498
RQLRGSQYQPILRDHICKDMSALVAARMRHIPLAPGSDWRDLPNIEVRLSDGTLARKLRYNYHDKKNGCSSTGALRGVCSCVEVGKACDPAARQFNTLIP 1491
RQLRGSQYQPILRDHICKDMSALVAARMRHIPLAPGSDWRDLPNIEVRLSDGTLARKLRYNYHDKKNGCSSSGALRGVCSCVE-GKPCEPAARQFNTLIP 1493
RQLRGSQYQPILRDHICKDMSALVAARMRHIPLAPGSDWRDLPNIEVRLSDGTMARKLRYTYHDRKNGRSSTGALRGVCSCVEAGKACDSAARQFNTLIP 1496
RQLRGSHYQPILRDHICKDMSALVAARMRHIPLSPGSDWRDLPNIQVRLRDGVITNKLRYTFHDTKNGCSSTGALRGVCSCAE-GKTCDPASRQFNTLIP 1500
RQLRGSHYQPILRDHICKDMSPLVAARMRHIPLFPGSDWRDLPNIQVRLGDGVIAHKLQYTFHDVKNGYSSTGALRGVCSCAE-GKACDPESRQFSTLIP 1498
RQIRGSQYQPILRDHICKDMSALVAGRMRHIPLAPGSDWRDLPNIEVRLSDGTTTRKLRYTHHEKKNGRSNTGALRGVCSCAE-GKPCDPADRQFNTLIP 1394
RQIRGSQYQPILRDHICKDMSALVAARMRHIPLAPGSDWRDLPNIEVRLRDGTTTKKLRYTHSDKKNGRSGTGALRGVCSCSE-GKQCDPADRQFNTLIP 1379

                                                                                                ←
WCLPHTGNRHNHWAGLYGRLEWDGFFSTTVTNPEPMGKQGRVLHPEQHRVVSVRECARSQGFPDTYRLFGNILDKHRQVGNAVPPPLAKAIGLEIRACVG 1511
WCLPHTGNRHNHWAGLYGRLEWDGFFSTTVTNPEPMGKQGRVLHPEQHRVVSVRECARSQGFPDTYRLFGNILDKHRQVGNAVPPPLAKAIGLEIKLCML 1613
--------RHGI---------------------------------------------------------------------------------------- 1502
WCLPHTGNRHNHWAGLYGRLEWDGFFSTTVTNPEPMGKQGRVLHPEQHRVVSVRECARSQGFPDTYRLFGNILDKHRQVGNAVPPPLAKAIGLEIKRCML 1591
WCLPHTGNRHNHWAGLYGRLEWDGFFSTTVTNPEPMGKQGRVLHPEQHRVVSVRECARSQGFPDTYRLFGNILDKHRQVGNAVPPPLAKAIGLEIKRCML 1593
WCLPHTGNRHNHWAGLYGRLEWDGFFSTTVTNPEPMGKQGRVLHPEQHRVVSVRECARSQGFPDTYRLFGNILDKHRQVGNAVPPPLAKAIGLEIKRCML 1596
WCLPHTGNRHNHWAGLYGRLEWDGFFSTTVTNPEPMGKQGRVLHPEQHRVVSVRECARSQGFPDTYRLFGNILDRHRQVGNAVPPPLAKAIGLEIKLCLL 1600
WCLPHTGNRHNHWAGLYGRLEWDGFFSTTVTNPEPMGKQGRVLHPEQHRVVSVRECARSQGFPDSYRFFGNILDRHRQVGNAVPPPLAKAIGLEIKLCLL 1598
WCLPHTGNRHNHWAGLYGRLEWDGFFSTTVTNPEPMGKQGRVLHPEQHRVVSVRECARSQGFPDTYRLFGNILDKHRQVGNAVPPPLAKAIGLEIKLCVL 1494
WCLPHTGNRHNHWAGLYGRLEWDGFFSTTVTNPEPMGKQGRVLHPEQHRVVSVRECARSQGFPDTYRFFGNVLDKHRQVGNAVPPPLSETIGLEVKKCVL 1479

ARMREESGAAVAPPAPEKMEMTAAAD 1537aa, chickenDNMT1
AKARESASAKIK--EEEAAKD----- 1632aa, humanDNMT1
-------------------------- 1502aa, chimpanzeeDNMT1
AKARESASVKVK--EEETTKD----- 1610aa, pigDNMT1
AKARESASAKIK--EEAAKD------ 1611aa, cattleDNMT1
AKAQESASVRVK--EEKTTAD----- 1615aa, horseDNMT1
ASAQESASAAVKGKEETTTED----- 1621aa, ratDNMT1
SSARESASAAVKAKEEAATKD----- 1619aa, mouseDNMT1
AKLKEN---ATEKIKQEHFESTD--- 1514aa, opossumDNMT1
EKMRENATEPVKQEKMELSD------ 1499aa, zebrafishDNMT1
```
