# Supplementary material for: Expression Patterns and miRNA Regulation of DNA Methyltransferases in Chicken Primordial Germ Cells
Source: PLoS One. 2011 May 3;6(5):e19524. doi: 10.1371/journal.pone.0019524 (PMC3086922; doi:10.1371/journal.pone.0019524)
Supplement: Figure S2 — Sequence comparison of vertebrate DNMT3A proteins. The protein sequences of DNMT3A from chicken, human, chimpanzee, pig, cattle, horse, rat, mouse, opossum, and zebrafish were aligned using the CLUSTAL X program and edited with the BioEdit program. Dark/light gray shaded sequences indicate amino acids identical/similar to those in chicken DNMT3A, and dashes represent gaps in the sequence. Arrows indicate the positions of the PWWP domain and DNA methylase domain in the chicken sequence. (PDF) [file pone.0019524.s002.pdf]

```
------------------------------------------------------------MVESSDTPKDTAAVPKCPPPCPEASPAEPLPNGDLEADGA 40
MPAMPSSGPG--DTSSSAAEREEDRKDGEEQEEPRGKEERQEPSTTARKVGRPGRKRKHPPVESGDTPKDPAVISKSPSMAQDSGASELLPNGDLEKRSE 98
---MPSSGPG--DTSSSAAEREEDRKDGEEQEEPRGKEERQEPSTTARKVGRPGRKRKHPPVESGDTPKDPAVISKSPSMAQDPGASELLPNGDLEKRSE 95
---------------------------------------------------------------------------------------------------- 1
--------------------------EGEEQEEARAKEERQEPSTTARKVGRPGRKRKHPPVESSDTPKDPAVTSKSLSMAQDSGPSELYPNGDLEKRSE 74
---MPSSGPG--DTSSSAPEREEDRKEGEEQEEARGKEERQEPSTAARKVGRPGRKRKHPPVESSDTPKDPTVTSKSPSMAQDSGSSELLPNGDLEKRSE 95
---MPSSGPG--DTSISSLEREDDRKEGEEQEENRGKEERQEPSATARKVGRPGRKRKHPPVESSDTPKDPAVTTKSQPTAQDSGPSDLLPNGDLEKRSE 95
---MPSSGPG--DTSSSSLEREDDRKEGEEQEENRGKEERQEPSATARKVGRPGRKRKHPPVESSDTPKDPAVTTKSQPMAQDSGPSDLLPNGDLEKRSE 95
---MPSSGATTVDNSSSSLERDEDRKEEEELEESKGKEERQDSSTPMRKVGRPGRKRKHPLVENSDTPKEVAPTSKAPSMAQDTGPSELLPNGDVEKPSE 97
----------------------------------------------------MNSMEDHGEVESEPKKDVITVPLLFPPPMPPTPPTPPMQ----QQPPQ 44

QWKGTEEGGASPKSGRPEFDETESLADGETGRALENGRCTPKEGLDAPADEGELAPSDPQKKRGRRKLLEATEKSKEEKEENNFDSLKMEGSRGRLRGGL 140
PQPEEGSPAGGQKGGAPAEGEGAAETLPEASRAVENGCCTPKEGRGAPAEAG-----------------------KEQKE-TNIESMKMEGSRGRLRGGL 174
PQPEEGSPAGGQKGGAPAEGEGAAETLPEASRAVENGCCTPKEGRGAPAEVG-----------------------KEQKE-TNIESMKMEGSRGRLRGGL 171
---------------------------------------------------------------------------------------------------- 1
PQPEEGSPAGGQKGGAPAEGEGATETPPEASRAVENGCCTPKDGRGAPAEEG-----------------------KEQKE-TNIESMKMEGSRGRLRGGL 150
PQPEEGSPAGGQKGGAPAEGEGAAETPTEASRAVENGCCTPKDGRGAPAEEG-----------------------KEQKE-TNIESMKMEGSRGRLRGGL 171
PQPEEGSPAAGQKGGAPAEGEG-TETPPEASRAVENGCCVTKEGRGASAGEG-----------------------KEQKQ-TNIESMKMEGSRGRLRGGL 170
PQPEEGSPAAGQKGGAPAEGEG-TETPPEASRAVENGCCVTKEGRGASAGEG-----------------------KEQKQ-TNIESMKMEGSRGRLRGGL 170
PQPEEGSPAG-RKGGARAEGEAEAESAPEAARTLENGCCTPKDGQEAPVVGG-----------------------KEEKEETNFDSVKMEGARGRLRGGL 173
PQAQH----------------------------------------------------------------------------------------------- 49

GWESSLRQRPMQRHTFQAGDPYYISKRKRDEWLARWKREAEKKAKVIAVMNVVEETP-RAEPQKEEEASPPASQQPTDPASPNVATTPEPVVADAVDKNT 239
GWESSLRQRPMPRLTFQAGDPYYISKRKRDEWLARWKREAEKKAKVIAGMNAVEENQGPGESQKVEEASPPAVQQPTDPASPTVATTPEPVGSDAGDKNA 274
GWESSLRQRPMPRLTFQAGDPYYISKRKRDEWLARWKREAEKKAKVIAGMNAVEENQGPGESQKVEEASPPAVQQPTDPASPTVATTPEPVGSDAGDKNA 271
-------------------------------------------------MNAMEENQGSTESQKVEEASPPAVQQPTDPASPTVATTPEPVGADAGDKNA 51
GWESSLRQRPMPRLTFQAGDPYYISKRKRDEWLARWKREAEKKAKVIAVMNAVEENQGSTESQKVEEASPPAVQQPTDPASPTVATTPEPVGADAGDKNA 250
GWESSLRQRPMPRLTFQAGDPYYISKRKRDEWLARWKREAEKKAKVIAVMNAVEENQGSGEAQKVEEASPPAVQQPTDPASPTVATTPEPVGADAGDKNV 271
GWESSLRQRPMPRLTFQAGDPYYISKRKRDEWLARWKREAEKKAKVIAVMNAVEESQASGESQKVEEASPPAVQQPTDPASPTVATTPEPVGADAGDKNA 270
GWESSLRQRPMPRLTFQAGDPYYISKRKRDEWLARWKREAEKKAKVIAVMNAVEENQASGESQKVEEASPPAVQQPTDPASPTVATTPEPVGGDAGDKNA 270
GWESSLRQRPMQRLTFQAGDPYYISKRKRDEWLARWKREAEKKAKVIAVMNVVEENQGPGEPQKVEEASPPTLQQPTDPASPTVATTPEPVGADAGDKNT 273
--QTNLKPQPSP----QQQPPPFQQQQQ---------------------------------------------QQPTDPASPTVATTPEPVSIGDGDKTS 98

                    PWWP domain
SKSADDEPEYEDGRGLGIGELVWGKLRGFSWWPGRIVSWWMTGRSRAAEGTRWVMWFGDGKFSVVCVEKLLPLSSFSSAFHQATYNKQPMYRKAIYEVLQ 339
TKAGDDEPEYEDGRGFGIGELVWGKLRGFSWWPGRIVSWWMTGRSRAAEGTRWVMWFGDGKFSVVCVEKLMPLSSFCSAFHQATYNKQPMYRKAIYEVLQ 374
TKAGDDEPEYEDGRGFGIGELVWGKLRGFSWWPGRIVSWWMTGRSRAAEGTRWVMWFGDGKFSVVCVEKLMPLSSFCSAFHQATYNKQPMYRKAIYEVLQ 371
TKSADDEPEYEDGRGFGIGELVWGKLRGFSWWPGRIVSWWMTGRSRAAEGTRWVMWFGDGKFSVVCVEKLMPLSSFCSAFHQATYNKQPMYRKAIYEVLQ 151
TKAADDEPEYEDGRGFGIGELVWGKLRGFSWWPGRIVSWWMTGRSRAAEGTRWVMWFGDGKFSVVCVEKLMPLSSFCSAFHQATYNKQPMYRKAIYEVLQ 350
TKAADDEPEYEDGRGFGIGELVWGKLRGFSWWPGRIVSWWMTGRSRAAEGTRWVMWFGDGKFSVVCVEKLMPLSSFCSAFHQATYNKQPMYRKAIYEVLQ 371
TKAADDEPEYEDGRGFGIGELVWGKLRGFSWWPGRIVSWWMTGRSRAAEGTRWVMWFGDGKFSVVCVEKLMPLSSFCSAFHQATYNKQPMYRKAIYEVLQ 370
TKAADDEPEYEDGRGFGIGELVWGKLRGFSWWPGRIVSWWMTGRSRAAEGTRWVMWFGDGKFSVVCVEKLMPLSSFCSAFHQATYNKQPMYRKAIYEVLQ 370
AKASDDEPEYEDGRGFGIGELVWGKLRGFSWWPGRIVSWWMTGRSRAAEGTRWVMWFGDGKFSVVCVEKLMPLSSFCSAFHQATYNKQPMYRKAIYEVLQ 373
PKS--TESEYEDGRGFGINELVWGKLRGYSWWPGRVVSWRMTRRSPAAEGTRWVTWFGDGKFSVVCVEKLLPLSSFHNAFHQPTYNKQPMYKKAIFEVLQ 196

VASSRAGKIFPACPENDETDTSKVVEIQNKQMIEWALGGFQPSGPKGLEPPEEERNPYKEVYTEMW--VEPEAAAYAPPPPAKKPRKS-TTEKPKVKEII 436
VASSRAGKLFPVCHDSDESDTAKAVEVQNKPMIEWALGGFQPSGPKGLEPPEEEKNPYKEVYTDMW--VEPEAAAYAPPPPAKKPRKS-TAEKPKVKEII 471
VASSRAGKLFPVCHDSDESDTAKAVEVQNKPMIEWALGGFQPSGPKGLEPPEEEKNPYKEVYTDMW--VEPEAAAYAPPPPAKKPRKS-TAEKPKVKEII 468
VASSRAGKLFPVCHDNDESDTAKAVEVQNKQMIEWALGGFQPSGPKGLEPPEEEKNPYKEVYTDMW--VEPEAAAYAPPPPAKKPRKS-TTEKPKVKEII 248
VASSRAGKLFPMCHDSDESDTAKAVEVQNKQMIEWALGGFQPSGPKGLEPPEEEKNPYKEVYTDMW--VEPEAAAYAPPPPAKKPRKS-TTEKPKVKEII 447
VASSRAGKLFPACHDNDESDTAKAVEVQNKQMIEWALGGFQPSGPKGLEPPEEEKNPYKEVYTDMW--VEPEAAAYAPPPPAKKPRKS-TTEKPKVKEII 468
VASSRAGKLFPACHDSDESDTGKAVEVQNKQMIEWALGGFQPSGPKGLEPPEEEKNPYKEVYTDMW--VEPEAAAYAPPPPAKKPRKS-TTEKPKVKEII 467
VASSRAGKLFPACHDSDESDSGKAVEVQNKQMIEWALGGFQPSGPKGLEPPEEEKNPYKEVYTDMW--VEPEAAAYAPPPPAKKPRKS-TTEKPKVKEII 467
VASSRAGKLFPACPENDESDTAKAVEVQNKQMIEWALGGFQPSGPKGLEPPEEERNPYKEVYTDMWAPVEPEAAAYAPPPPAKKPRKS-TAEKPKIKEII 472
VASTRAGKTFLSCPASDDTDSSKLVDVENKKMIEWAMSGFQPTGPKGLDPPESE--PY-----EMCEWVEPEAASYLPPP-AKKPRKSTVTEKPKVKEMI 288

DERTRERLVYEVRQKCRNIEDICISCGSLNVTLEHPLFIGGMCQNCKNCFLECAYQYDDDGYQSYCTICCGGREVLMCGNNNCCRCFCVECVDLLVGPGA 536
DERTRERLVYEVRQKCRNIEDICISCGSLNVTLEHPLFVGGMCQNCKNCFLECAYQYDDDGYQSYCTICCGGREVLMCGNNNCCRCFCVECVDLLVGPGA 571
DERTRERLVYEVRQKCRNIEDICISCGSLNVTLEHPLFVGGMCQNCKNCFLECAYQYDDDGYQSYCTICCGGREVLMCGNNNCCRCFCVECVDLLVGPGA 568
DERTRERLVYEVRQKCRNIEDICISCGSLNVTLEHPLFIGGMCQNCKNCFLECAYQYDDDGYQSYCTICCGGREVLMCGNNNCCRCFCVECVDLLVGPGA 348
DERTRERLVYEVRQKCRNIEDICISCGSLNVTLEHPLFIGGMCQNCKNCFLECAYQYDDDGYQSYCTICCGGREVLMCGNNNCCRCFCVECVDLLVGPGA 547
DERTRERLVYEVRQKCRNIEDICISCGSLNVTLEHPLFIGGMCQNCKNCFLECAYQYDDDGYQSYCTICCGGREVLMCGNNNCCRCFCVECVDLLVGPGA 568
DERTRERLVYEVRQKCRNIEDICISCGSLNVTLEHPLFIGGMCQNCKNCFLECAYQYDDDGYQSYCTICCGGREVLMCGNNNCCRCFCVECVDLLVGPGA 567
DERTRERLVYEVRQKCRNIEDICISCGSLNVTLEHPLFIGGMCQNCKNCFLECAYQYDDDGYQSYCTICCGGREVLMCGNNNCCRCFCVECVDLLVGPGA 567
DERTRERLVYEVRQKCRNIEDICISCGSLNVTLEHPLFIGGMCQNCKNCFLECAYQYDDDGYQSYCTICCGGREVLMCGNNNCCRCFCVECVDLLVGPGA 572
DERTRERLVFDVRQKCRNIEDICISCGSLYVSLEHPLFIGGMCQSCTNCFLECAYQYDDDGYQSYCTICCGGREVLMCGNNNCCRCFCVECVDLLVGPGA 388

                                                                             DNA Methylase domain
AQAAIKEDPWNCYMCGHKGVYGLLRRREDWPSRLQMFFANNHDQEFDPPKVYPPVPAEKRKPIRVLSLFDGIATGLLVLKDLGIQVDRYIASEVCEDSIT 636
AQAAIKEDPWNCYMCGHKGTYGLLRRREDWPSRLQMFFANNHDQEFDPPKVYPPVPAEKRKPIRVLSLFDGIATGLLVLKDLGIQVDRYIASEVCEDSIT 671
AQAAIKEDPWNCYMCGHKGTYGLLRRREDWPSRLQMFFANNHDQEFDPPKVYPPVPAEKRKPIRVLSLFDGIATGLLVLKDLGIQVDRYIASEVCEDSIT 668
AQAAIKEDPWNCYMCGHKGTYGLLRRRDDWPSRLQMFFANNHDQEFDPPKVYPPVPAEKRKPIRVLSLFDGIATGLLVLKDLGIQVDRYIASEVCEDSIT 448
AQAAIKEDPWNCYMCGHKGTYGLLRRRDDWPSRLQMFFANNHDQEFDPPKVYPPVPAEKRKPIRVLSLFDGIATGLLVLKDLGIQVDRYIASEVCEDSIT 647
```

```
AQAAIKEDPWNCYMCGHKGTYGLLRRRDDWPSRLQMFFANNHDQEFDPPKVYPPVPAEKRKPIRVLSLFDGIATGLLVLKDLGIQVDRYIASEVCEDSIT 668
AQAAIKEDPWNCYMCGHKGTYGLLRRREDWPSRLQMFFANNHDQEFDPPKVYPPVPAEKRKPIRVLSLFDGIATGLLVLKDLGIQVDRYIASEVCEDSIT 667
AQAAIKEDPWNCYMCGHKGTYGLLRRREDWPSRLQMFFANNHDQEFDPPKVYPPVPAEKRKPIRVLSLFDGIATGLLVLKDLGIQVDRYIASEVCEDSIT 667
AQAAIKEDPWNCYMCGHKGTYGLLRRRDDWPSRLQMFFANNHDQEFDPPKVYPPVPAEKRKPIRVLSLFDGIATGLLVLKDLGIQVDRYIASEVCEDSIT 672
AQAAIKEDPWNCYMCGLKTQYGLLERRADWPCRLQHFFANNHDQEFEPPRLYPPVLAENRKPIRVLSLFDGIATGLLVLRELGIQVERYVASEVCEDSIT 488

VGMVRHQGKIMYVGDVRNVTQKHIQEWGPFDLVIGGSPCNDLSIVNPARKGLYEGTGRLFFEFYRLLHEARPKEGDDRPFFWLFENVVAMGVSDKRDISR 736
VGMVRHQGKIMYVGDVRSVTQKHIQEWGPFDLVIGGSPCNDLSIVNPARKGLYEGTGRLFFEFYRLLHDARPKEGDDRPFFWLFENVVAMGVSDKRDISR 771
VGMVRHQGKIMYVGDVRSVTQKHIQEWGPFDLVIGGSPCNDLSIVNPARKGLYEGTGRLFFEFYRLLHDARPKEGDDRPFFWLFENVVAMGVSDKRDISR 768
VGMVRHQGKIMYVGDVRSVTQKHIQEWGPFDLVIGGSPCNDLSIVNPARKGLYEGTGRLFFEFYRLLHDARPKEGDDRPFFWLFENVVAMGVSDKRDISR 548
VGMVRHQGKIMYVGDVRSVTQKHIQEWGPFDLVIGGSPCNDLSIVNPARKGLYEGTGRLFFEFYRLLHDARPKEGDDRPFFWLFENVVLGVSDKRDISR 747
VGMVRHQGKIMYVGDVRSVTQKHIQEWGPFDLVIGGSPCNDLSIVNPARKGLYEGTGRLFFEFYRLLHDARPKEGDDRPFFWLFENVVAMGVSDKRDISR 768
VGMVRHQGKIMYVGDVRSVTQKHIQEWGPFDLVIGGSPCNDLSIVNPARKGLYEGTGRLFFEFYRLLHDARPKEGDDRPFFWLFENVVAMGVSDKRDISR 767
VGMVRHQGKIMYVGDVRSVTQKHIQEWGPFDLVIGGSPCNDLSIVNPARKGLYEGTGRLFFEFYRLLHDARPKEGDDRPFFWLFENVVAMGVSDKRDISR 767
VGMVRHQGKIMYVGDVRNVTQKHIQEWGPFDLVIGGSPCNDLSIVNPARKGLYEGTGRLFFEFYRLLHEARPKEGDDRPFFWLFENVVAMGVSDKRDISR 772
VGIVRHQGRIMYVGDVRQLTRKNIQEWGPFDLVIGGSPCNDLSIVNPARKGLYEGTGRLFFEFYRLLHEARPKEGDTRPFFWLFENVVAMGVSDKRDISR 588

FLESNPVMIDAKEVSAAHRARYFWGNLPGMNRPLASTVNDKLELQECLEHGRIAKFSKVRTITTRSNSIKQGKDQHFPVFMNEKEDILWCTEMERVFGFP 836
FLESNPVMIDAKEVSAAHRARYFWGNLPGMNRPLASTVNDKLELQECLEHGRIAKFSKVRTITTRSNSIKQGKDQHFPVFMNEKEDILWCTEMERVFGFP 871
FLESNPVMIDAKEVSAAHRARYFWGNLPGMNRPLASTVNDKLELQECLEHGRIAKFSKVRTITTRSNSIKQGKDQHFPVFMNEKEDILWCTEMERVFGFP 868
FLESNPVMIDAKEVSAAHRARYFWGNLPGMNRPLASTVNDKLGLQECLEHGRIAKFSKVRTITTRSNSIKQGKDQHFPVFMNEKEDILWCTEMERVFGFP 648
FLESNPVMIDAKEVSAAHRARYFWGNLPGMNRPLASTVNDKLELQECLEHGRIAKFSKVRTITTRSNSIKQGKDQHFPVFMNEKEDILWCTEMERVFGFP 847
FLESNPVMIDAKEVSAAHRARYFWGNLPGMNRPLASTVNDKLELQECLEHGRIAKFSKVRTITTRSNSIKQGKDQHFPVFMNEKEDILWCTEMERVFGFP 868
FLESNPVMIDAKEVSAAHRARYFWGNLPGMNRPLASTVNDKLELQECLEHGRIAKFSKVRTITTRSNSIKQGKDQHFPVFMNEKEDILWCTEMERVFGFP 867
FLESNPVMIDAKEVSAAHRARYFWGNLPGMNRPLASTVNDKLELQECLEHGRIAKFSKVRTITTRSNSIKQGKDQHFPVFMNEKEDILWCTEMERVFGFP 867
FLESNPVMIDAKEVSAAHRARYFWGNLPGMNRPLASTVNDKLELQECLEHGRIAKFSKVRTITTRSNSIKQGKDQHFPVFMNEKEDILWCTEMERVFGFP 872
FLECNPVMIDAKEVSAAHRARYFWGNLPGMNRPMSAMCTDKLDLQDCLEHGRTAKFGKVRTITTRSNSIKQGKDQHFPVFMNDKEDILWCTEMERVFGFP 688

VHYTDVSNMSRLARQRLLGRSWSVPVIRHLFAPLKEYFACV-- 877aa, chickenDNMT3A
VHYTDVSNMSRLARQRLLGRSWSVPVIRHLFAPLKEYFACV-- 912aa, humanDNMT3A
VHYTDVSNMSRLARQRLLGRSWSVPVIRHLFAPLKEYFACV-- 909aa, chimpanzeeDNMT3A
VHYTDVSNMSRLARQRLLGRSWSVPVIRHLFAPLKEYFACV-- 689aa, pigDNMT3A
VHYTDVSNMSRLARQRLLGRSWSVPVIRHLFAPLKEYFACV-- 888aa, cattleDNMT3A
VHYTDVSNMSRLARQRLLGRSWSVPVIRHLFAPLKEYFACV-- 909aa, horseDNMT3A
VHYTDVSNMSRLARQRLLGRSWSVPVIRHLFAPLKEYFACV-- 908aa, ratDNMT3A
VHYTDVSNMSRLARQRLLGRSWSVPVIRHLFAPLKEYFACV-- 908aa, mouseDNMT3A
VHYTDVSNMSRLARQRLLGRSWSVPVIRHLFAPLKEYFACV-- 913aa, opossumDNMT3A
VHYTDVSNMSRLARQRLLGRSWSVPVIRHLFAPLKEYFACVGT 731aa, zebrafishDNMT3A
```
