# Supplementary material for: Expression Patterns and miRNA Regulation of DNA Methyltransferases in Chicken Primordial Germ Cells
Source: PLoS One. 2011 May 3;6(5):e19524. doi: 10.1371/journal.pone.0019524 (PMC3086922; doi:10.1371/journal.pone.0019524)
Supplement: Figure S3 — Sequence comparison of vertebrate DNMT3B proteins. The protein sequences of DNMT3B from chicken, human, chimpanzee, pig, cattle, horse, rat, mouse, opossum, and zebrafish were aligned using the CLUSTAL X program and edited with the BioEdit program. Dark/light gray shaded sequences indicate amino acids identical/similar to those in chicken DNMT3B, and dashes represent gaps in the sequence. Arrows indicate the positions of the PWWP domain and DNA methylase domain in the chicken sequence. (PDF) [file pone.0019524.s003.pdf]

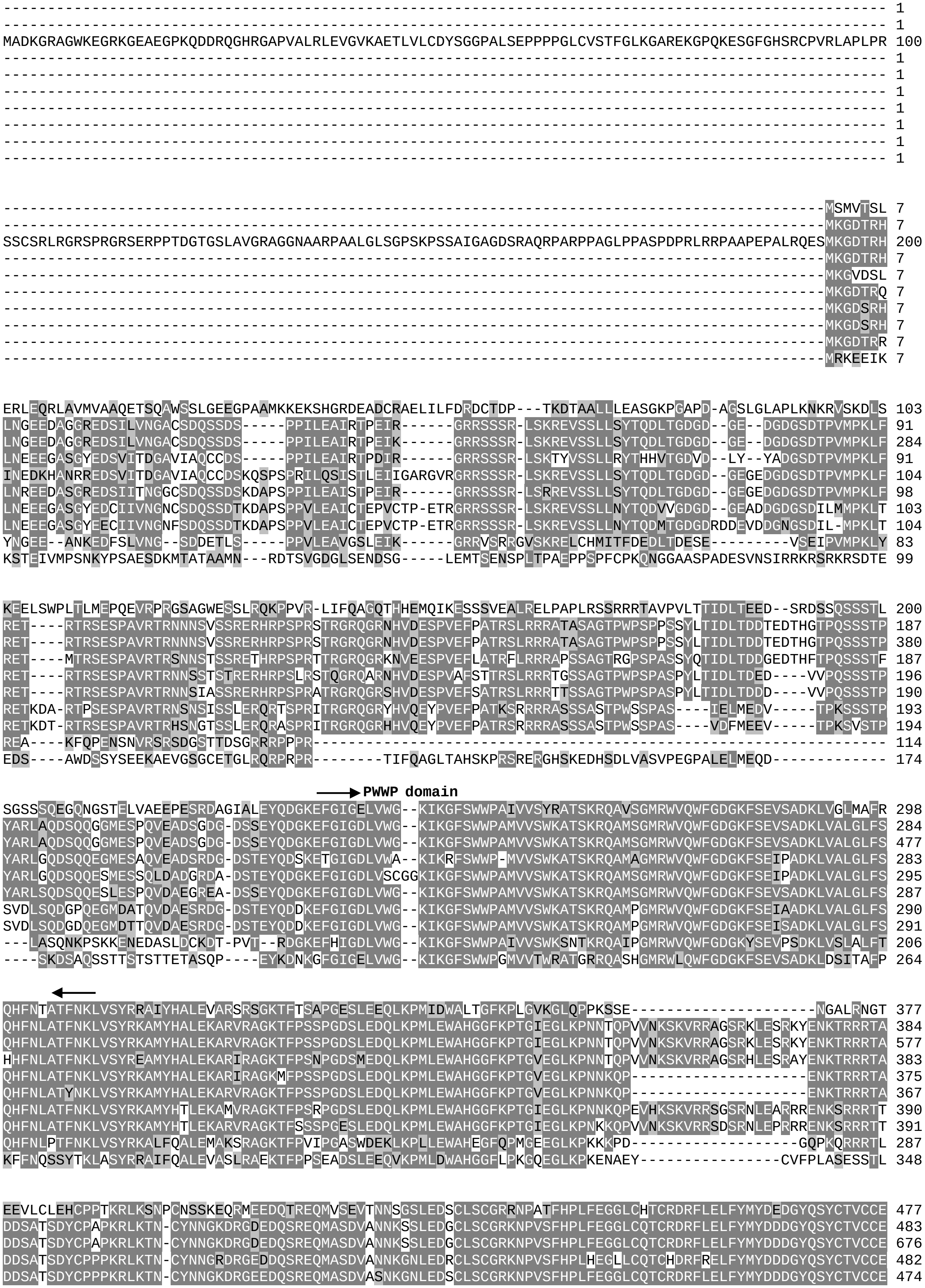
PWWP domain

```
DDSAASDYCPPPKRLKTN-CYNNGKDRGEEDQSREQMALDVTNNKSSLEDSCLSCGRKNPVSFHPLFEGGLCQTCRDRFLELFYMYDDDGYQSYCTVCCE 466
IDFAASEYSTPPKRLKTN-SYG-GKDRGEDEESRERMASDVTNNKGNLEDRCLSCGKKNPVSFHPLFEGGLCQSCRDRFLELFYMYDEDGYQSYCTVCCE 488
NDSAASE-SPPPKRLKTN-SYG-GKDRGEDEESRERMASEVTNNKGNLEDRCLSCGKKNPVSFHPLFEGGLCQSCRDRFLELFYMYDEDGYQSYCTVCCE 488
EEVTVQDSLPPPKRLKPS-CVNNNKERVEEDQSREKMVSDLKNNKSNPEDSCIACGRRNPPSFHPLFEGGLCHTCRDRFLELFYMYDDDGYQSYCTVCCE 386
LESSPPEFPPSAKRARLP--LNKAKPGIEEVYSREQMVNEVLKNHRSIEEFCLSCGKTRVATFHPLFEGGLCLTCKDAYLENSYMYDDDGYQSYCTVCCG 446

GKELLLCSNASCCRCFCVECLEVLVGRGSSAKAKEQEPWNCYMCQPQRSYGVLQRXQDWSSRLQDFFTSDK-GQEYDAPKIYPAVPPAKRRPIRVLSLFD 576
GRELLLCSNTSCCRCFCVECLEVLVGTGTAAEAKLQEPWSCYMCLPQRCHGVLRRRKDWNVRLQAFFTSDT-GLEYEAPKLYPAIPAARRRPIRVLSLFD 582
GRELLLCSNTSCCRCFCVECLEVLVGTGTAAEAKLQEPWSCYMCLPQRCHGVLRRRKDWNVRLQAFFTSDT-GLEYEAPKLYPAIPAARRRPIRVLSLFD 775
GRELLLCSTTSCCRCLCVECLEVLVGTGTAAEAKLQEPWSCYMCLSQRCHPVLRRRKDWNVRLQAFGTSDT-LLEYEDSKLYPAIPAARRRPIRVLSLLD 581
GRELLLCSNTSCCRCFCVECLEVLVGAGTAAEAKLQEPWSCYMCLPQRCHGILRRRKDWSVRLQAFFTSDP-GLEYEAPKLYPAIPANRRRPIRVLSLFD 573
GRELLLCSNTSCCRCFCVECLEVLVGSGTAAHAKLQEPWSCYMCLPQRCHGVLRRRKDWNVRLQAFFTSDT-GLEYEAPKLYPAIPAARRRPIRVLSLFD 565
GRELLLCSNTSCCRCFCVECLEVLVGTGTAEDAKLQEPWSCYMCLPQRCHGVLRRRKDWNMRLQDFFTTDPDLEEFEPPKLYPAIPAAKRRPIRVLSLFD 588
GRELLLCSNTSCCRCFCVECLEVLVGAGTAEDAKLQEPWSCYMCLPQRCHGVLRRRKDWNMRLQDFFTTDPDLEEFEPPKLYPAIPAAKRRPIRVLSLFD 588
GRELLLCSNTSCCRCFCVECLDVLVGAGTSASAKAQEPWSCYMCLPQRCYGILQRRKDWNIRLQEFFTNDI-AQEYAVPKLYPAVPASRRRPIRVLSLFD 485
GREMLLCGNANCCRCICVDCLDILVGAGAANSARNLDPWRCYMCQPLQQYGVLKKRHDWSLKLQEFFVNDS-GQEFESPKIYPAVPAEQRRPIRVLSLFD 545
```

**DNA Methylase domain**

```
GIATGYLVLKDLGIQVEKYIASEICEDPLAVGTVRHEGNITYVH--DVRNITKRNIEEWGPFDLVIGGSPCNDLSLASPPRKAPYGEAAWPHGAGSPQAA 674
GIATGYLVLKELGIKVGKYVASEVCEESIAVGTVKHEGNIKYVN--DVRNITKKNIEEWGPFDLVIGGSPCNDLSNVNPARKGLY--------------- 665
GIATGYLVLKELGIKVGKYVASEVCEESIAVGTVKHEGNIKYVN--DVRNITKKNIEEWGPFDLVIGGSPCNDLSNVNPARKGLY--------------- 858
GIATGYLVLKELGIKVGKYVASEVCEESIAVGTVKHEGNIKYVN--DVRNITKKNIEEWGPFDLVIGGSPCNDLSNVNPARKGLY--------------- 664
GIATGYLVLKELGIKVEKYVASEVCEESIAVGTVKHEGNIKYVN--DVRNITKKNIEEWGPFDLVIGGSPCNDLSNVNPARKGLY--------------- 656
GIATGYLVLKELGIKVK-YVASEVCEESIAVGSLKHERQLSKXCXMTSRDITKRNIEEWGPFDLVIGGSPCNDLSNVNPARKGLY--------------- 649
GIATGYLVLKDLGIKVEKYVASEVCAESIAVGTIKHEGQIKYVN--DVRKITKKNIEEWGPFDLVIGGSPCNDLSNVNPARKGLY--------------- 671
GIATGYLVLKELGIKVEKYIASEVCAESIAVGTVKHEGQIKYVN--DVRKITKKNIEEWGPFDLVIGGSPCNDLSNVNPARKGLY--------------- 671
GIATGYLVLKELGIKVDKYIASEICEDSIAVGTVKHEGNIKYVH--DVRNITKRQIDEWGPFDLLIGGSPCNDLSNVNPARKGLY--------------- 568
GIATGYLVLRDLGFKVDLYIASEVCEDSISVGAVRHEGKIQYVH--DVRNITRKNIAEWGPFDMVIGGSPCNDLSIVNPARKGLY--------------- 628

WGRRRSCAVAPSGDTCQALSPPGPPEPVPTPQPNLRGRGRSPPNTPAGSPRTEGTGRLFFEFYHLLNYARPKAGEERPFFWMFENVVAMRVNDKRDISRF 774
-------------------------------------------------------EGTGRLFFEFYHLLNYSRPKEGDDRPFFWMFENVVAMKVGDKRDISRF 713
-------------------------------------------------------EGTGRLFFEFYHLLNYSRPKEGDDRPFFWMFENVVAMKVGDKRDISRF 906
-------------------------------------------------------EGTGRLFFEHYHLLNYSRSKEGDDRPFFWMFENVVAMQVGDKRDISRF 712
-------------------------------------------------------EGTGRLFFEFYHLLNYTRPKEGEDRPFFWMFENVVAMKVGDKRDISRF 704
-------------------------------------------------------EGTGRLFFEFYHLLNYSRPKEGDDRPFFWMFENVVAMKVGDKRDISRF 697
-------------------------------------------------------EGTGRLFFEFYHLLNYTRPKEGDNRPFFWMFENVVAMKVNDKKDISRF 719
-------------------------------------------------------EGTGRLFFEFYHLLNYTRPKEGDNRPFFWMFENVVAMKVNDKKDISRF 719
-------------------------------------------------------EGTGRLFFEFYHLLNYTRPKEGDERPFFWMFENVVAMRVNDKRDISRF 616
-------------------------------------------------------EGTGRLFFEFYRLLSEAKPKEGEDRPFFWMFENVVAMSVNDKRDISRF 676

LECNPVMIDAIKISAAHRARYFWGNLPGMN---------------------------------------------------------------------RIFGFPL 811
LECNPVMIDAIKVSAAHRARYFWGNLPGMNRPVIASKNDKLELQDCLEYNRIAKLKKVQTITTKSNSIKQGKNQLFPVVMNGKEDVLWCTELERIFGFPV 813
LECNPVMIDAIKVSAAHRARYFWGNLPGMNRPVIASKNDKLELQDCLEYNRIAKLKKVQTITTKSNSIKQGKNQLFPVVMNGKEDVLWCTELERIFGFPV 1006
LECNPVMIDAIKVSAAHRARYFWGNLPGMNRSVIASTNDSLELQDCLEYNRIAKLKKVQTITTKSNSIKQGKNQLFPVVMNGKEDVLWCTELERIFGFPV 812
LECNPVMIDAIKVSAAHRARYFWGNLPGMNRPVIASKNDKLELQDCLEFNRTAKLKKVQTITTKSNSIRQGKNQLFPVVMNGKEDVLWCTELERIFGFPV 804
LECNPVMIDAIKVSAAHRARYFWGNLPGMN---------------------------------------------------------------------RIFGFPV 734
LACNPVMIDAIKVSAAHRARYFWGNLPGMNRPVIASKNDKLELQDCLEFSRTAKLKKVQTITTKSNSIRQGKNQLFPVVMNGKDDVLWCTELERIFGFPA 819
LACNPVMIDAIKVSAAHRARYFWGNLPGMNRPVMASKNDKLELQDCLEFSRTAKLKKVQTITTKSNSIRQGKNQLFPVVMNGKDDVLWCTELERIFGFPA 819
LECNPVMIDAIKVSAAHRARYYWGNLPGMNRPVIASKTDKLELQDCLEYNRIAKLKKVQTITTKSNSIKQGKNQQFPVIMNGKEDILWCTELERIFGFPV 716
LECNPVMIDAIEVSAAHRARYFWGNLPGMKRPLCASGMDKLELQDCLEHGRVAKFGKVRTITTRSNSIKQGKDQHFPVMMNGKEDILWCTELERIFGFPV 776

HCTDVSNISRGARQKLLGRSWSIPVIQHLFSPLKDYFACE 851aa, chickenDNMT3B
HYTDVSNMGRGARQKLLGRSWSVPVIRHLFAPLKDYFACE 853aa, humanDNMT3B
HYTDVSNMGRGARQKLLGRSWSVPVIRHLFAPLKDYFACE 1046aa,chimpanzeeDNMT3B
HYTDVSNMGRVARQKLPGRSWSVPVIRHLFAPRKDYFACE 852aa, pigDNMT3B
HYTDVSNMGRVARQKLLGRSWSVPVIRHLFAPLKDYFACE 844aa, cattleDNMT3B
HYTDVSNMGRGARQKLLGRSWSVPVIRHLFAPLKDYFACE 774aa, horseDNMT3B
HYTDVSNMGRGARQKLLGRSWSVPVIRHLFAPLKDYFACE 859aa, ratDNMT3B
HYTDVSNMGRGARQKLLGRSWSVPVIRHLFAPLKDYFACE 859aa, mouseDNMT3B
HYTDVSNMGRGARQKLLGRSWSVPVIRHLFAPLKDYFACE 756aa, opossumDNMT3B
HYTDVSNMGRGARQKLLGRSWSVPVIRHLFAPLKDYFACE 816aa, zebrafishDNMT3B
```
