# Supplementary material for: Expression Patterns and miRNA Regulation of DNA Methyltransferases in Chicken Primordial Germ Cells
Source: PLoS One. 2011 May 3;6(5):e19524. doi: 10.1371/journal.pone.0019524 (PMC3086922; doi:10.1371/journal.pone.0019524)
Supplement: Figure S4 — Comparison of chicken DNMT3B 3′UTR and DNMT3B 3′UTR specific miRNA binding sites with human and mouse DNMT3B 3′UTR using the CLUSTAL X program. miR-15c, miR-29b, miR-383 and miR-222 binding sites in chicken and corresponding human and mouse sequences are shown in red colour. (PDF) [file pone.0019524.s004.pdf]

```
-------------------------------------------------------------CCCACGCTGCATCCCTCGTCTCTCCGTCCCAACGCAGCT 39
TTCCAGCCAGGCCCCAAGCCCACTGGGGTGTGTGGCAGAGCCAGGACCCAGGAGGTGTGATTCCTGAAGGCATCCC--CAGGCCCTGCTCTTCCTCAGCT 98
TTCTACCCAGGACTGGGGAGCTCTCG-------GTCAGAGCCAGTGCCCAGAGTCACCCCTCCCTGAAGGCACCTCACCTGTCCCCTTTTTAGCTCACCT 93

CTGCTG-------CCCGGGGAGGGCTCAGCCCTGTGCCCACCATTCCCAGCACTGTGCCCCCGCTGCCGCAGGG-----CTCGGGAAGCGCCTTCAACCA 127
GTGTGGG---TCATACCG-TGTACCTCAGTTCCCTCTTGCTCAGTGGGGGCAGAGCCACCTGACTCTTGCAGGGGTAGCCTGAGGTGCCGCCTCCTTGTG 194
GTGTGGGGCCTCACATCACTGTACCTCAGCTTTCTCCTGCTCAGTGGGAGCAGAGCCTCCTGGCCCTTGCAGGGGAGCCCC--GGTGCTCCCTCCGTGTG 191

AAGAACCTGG--CCGGGTGCCTT------CCTGACAGGGACTACAGCCAGCGCTGCCTGGGAAATGATGGAC--AAAACGAGCTTTAAT-CACCCAGCAC 216
CACAAATCAGACCTGGCTGCTTGGAGCAGCCTAACACGGTGCTCATTTTTTCTTCTCCTAAAACTTTAAAACTTGAAGTAGGTAGCAACGTGGCTTTTTT 294
CACAGCTCAGACCTGGCTGCTTAGAGTAGCCCGGCATGGTGCTCATGTTCTCTTACCCTGAAACTTTAAAACTTGAAGTAGGTAGTAAGATGGCTTTCTT 291

AAAGCTGCGTGCATCACTGATGAGGC-GAGAAGTCTCCTCCAGAGCATTAAACATTCCACTTGAGCCCAGAGCTGGGAAGCAGTGAGTGGTGGAGGCAGC 315
TTTTTCCCTTCCTGGGTCTACCACTCAGAGAAACAATGGCTAAGATACCAAA-ACCACAG-TGCCGACAGCTCTCCAATACTCAGGTTAATGCTGAAAAA 392
T---TACCCTCCTGAGTTTATCACTCAGA--AGTGATGGCTAAGATACCAAA-AAAACAAACAAAAACAGAAACAAAAAACAAAAAAAAAACCTCAACAG 385

TGCTGGCGTGCTGCAAATGTGGACTGCA-GAGCTGCTCACCTCCTAAA------CAAGCATAAACCTCTCTTGA--AACGACTGCCA--AAAACCCTCAA 404
TCATCCAAGA---CAGTT----ATTGCAAGAG---TTTAATTTTTGAA---AACTGGCTACTGCTCTGTGTTTACAGACGTGTGCAGTTGTAGGCATGTA 479
CTCTCTTAGTACTCAGGTTCATGCTGCAAAATCACTTGAGATTTTGTTTTTAAGTAACCCGTGCTCCACATTTGCTGGAGGATGCTATTGTGAATGTGGG 485

GCTGCAGCAGCAGGTGTGAGGGCCCCAGGTCAGGTTATGTACACTGAACGTGCAGAACACACAA-ACCAAATCTTTTTT---TTTGTTTTTGATTTTTAA 500
GCTACAGGA-CATTTTTAAGGGCCCAGGATCGTTTTTTCC---CAGGGCAAGCAGAAGAGAAAATGTTGTATATGTCTT----TTACCCGGCACATTCCC 571
CTCAGATGAGCAAGGTCAAGGGGCCAAAAAAAATTCCCCCTCTCCCCCCAGGAGTATTTGAAGATGATGTTTATGGTTTAAGTCTTCCTGGCACCTTCCC 585
                                                                                  miR-15c
GACCTTTTTGTAAAACTTTTACATTTTG---------TTGTTTAACGTCTATTGCTGCTTTAAGGCATTACATACGAATGTGGAGAC-------TCCTAG 584
CTTGCCTAAATACAAGGGCTGGAGTCTGCACGGGACCTATTAGAGTATTTTCCACAATGATGATGATTTCAGCAGGGATGACGTCATCATCACATTCAGG 671
CTTGCTTTGGTACAAGGGCTGAAGTCCTGTTGG----TCTTGTAGCATTTCCCAGGATGATGATG---TCAGCAGGGATGACATCATCACC---TTTAGG 675
        miR-222
ACCTGAATGTAGCT-----------GAGACTGAACTGTGAGCTGATAGAATTACTTTTCTAGTTAGGGAGATACTGTTTTATGTGTTCAGCTGTAG-TTT 672
GCTATTTTTTCCCCCACAAACCCAAGGGCAGGGGCCACTCTTAGCTAAATCCCTCCCCGTGACTGCAATAGAACCCTCTGGGGAGCTCAGGAAGGGGTGT 771
GCT----TTTCCCT------------GGCAGGGGCC-CATGTGGCTAG--TCCTCACGAAGACTGGAGTAGAATGTTT---GGAGCTCAGGAAGGG-TGG 752
                                                                                             miR-15c
ACGGATATTCACTACGTCCTTTTCCA--------------------GGCATTTCCCTTTTTTCTCCTGACGTTTCAAAATGCTGCTGAGGGGGTTGGATG 752
GCTGAGTTCTATAATATAAGCTGCCATATATTTTGTAGACAAGTATGGCTCCTCCATATCTCCCTCTTCCCTAGGAGAGGAGTGTGAAGCAAGGAGCTTA 871
GTGGAGT---------------------------------------GGC-------------CCTCTTCCAGGTGTGAGGGATACGAAGGAGGAAGCTTA 800

GTTACACTGGGGCAGGGAAATGGGGCTGTTCTGAGAATGAGTGCATGGGGTGGGCAGGGGTGGGCCGCTGTTG---TCCCCCCCCWCCCCCCCCGTGCTC 849
GATAAGACACCCCCTCAAACCCATTCCCTCTCCAGGAGACCTACCCTCCACAGGCACAGGTCCCCAGATGAGAAGTCTGCTACCCTCATTTCTCATCTTT 971
GGGAAATCCATTCCCCACTCCCTCTTGCCAAATGAGGGGCCCAGTCCCCAACAGCTCAGGTCCCCAGAA-----------CCCCCTAGTTCCTCATG--- 886

TCACCCAGCA-GCAGCCCAGGACTGTGGGTGCCACCGGGCTGCCCACCCCTCTGCCACCCCATTCTATGAGTTTTGTTCCTTGGAGC----CACGTGGGC 944
TTACTAAACTCAGAGGCAGTGACAGCAGTCAGGGACAGACATACATTTCTCATACCTTCCCCACATCTGAGAGATGACAGGGAAAAC-TGCAAAGCTCGG 1070
-----------AGAAGCTAGGAC------CAGAAGCACAT---CGTTCCCCTTATCTGAGCAGTGTTTGGGGAACTACAGTGAAAACCTTCTGGAGATGT 966
                                                                                 miR-383
TCCCCTCAGTCGC-ATCCTCTGTTTCATGTGCTGGATCTCACCTCCCTTCAGTCTGATCAAAAGGAAAAGAGAGAAAGAAAGGACTCGGGGAAAGCAAAC 1043
TGCTCCCTTTGGAGATTTTTTAATCCTTTTTTATTCCATAAGAAGTCGTTTTTAGGGAGA--ACGGGAATTCAGACAAGCTGCATTTCAGAAATGCTGTC 1168
TAAAAGCTTTTT--ACCCCACGATAGATTGTGTTTTTAAGGGGTGCTTTTTTTAGGGGCATCACTGGAGATAAGA-AAGCTGCATTTCAGAAATGCCATC 1063

A-AAGGATAAAAGGGGAMCAAGAACGGTCCTBGCGTTAAGCTGACTTGATGAGAATCGCAGTA---GGACAAGTCTT-------TCCTTGGA-------- 1124
ATAATGGTTTTTAA-CACCTTTTACTCTTCTTAC-TGGTGCTATTTTGTAGAATAAGGAACAACGTTGACAAGTTTTGTGGGGCTTTTTATACACTTTTT 1266
GTAATGGTTTTTAAACACCTTTTAC-CTAATTAC-AGGTGCTATTTTATAGAA-----------GCAGACAACACTT------CTTTTTATG-------- 1136

--ACGCT---GCCTTTCATGGTGTGCTCATCAACM-CATTGCAAGGAGAGGTACAGCTCAGAAATGCCTTTTTTTAATGCTTTGTAAAGGTTTCTAACTC 1218
AAAATCTCAAACTTCTATTTTTATGTTTAACGTTTTCATTAAAATTTTTTTTGTAACT-GGAGCCACGACGTAACAAATATGGGGAAAAAACTGTGCCTT 1365
--ACTCTCAGACTTCTATTTTCATGTT-ACCATTTTTTTTGTAACTCGCAAGGTGTGG-GCTTTTGTAACTTCACAGGTGTGGGGAGAGAC---TGCCTT 1229
     miR-29b
TCCCTGGTGCTATTTTTTTAGT--TAAAGGATTTTTGATGTCCAAAGTTTTGTGGGTTTTTATACAATCTTTTAAAATAAGAGRAAAAAAAAAAAGCCAA 1316
GTTTCAACAGTTT-----------TTGCTAATTTTTAGGCTGAAAGATGACGGATGCCTAGAGTTTACCTTA-TGTTTAATTAAAATCAGTATTTGTCTA 1453
GTTTCAACAGTTTGTCTCCACTGGTTTCTAATTTTTAGGTGCAAAGATGACAGATGCCCAGAGTTTACCTTTCTGGTTGATTAAAGTT-GTATTTCTCTA 1328

AAAAAAAAAAAAAAAAAAACCGAGGGGGGTCA 1348, Chicken
AAAAAAAAAAAAAAAAA--------------- 1470, Human
TAAAAAAAAAA--------------------- 1339, Mouse
```
